# Microstructural and physicochemical characterization, mechanical and durability performance with statistical evaluation of montmorillonite-impregnated burnt red soil bricks incorporating pyrolyzed coffee and tobacco biochars

**DOI:** 10.1371/journal.pone.0357232

**Published:** 2026-09-21

**Authors:** Amani Abdallah Hepautwa, Askwar Hilonga, Register Mrosso, Tusekile Alfredy, Yusufu Abeid Chande Jande

**Affiliations:** 1 Department of Materials and Energy Sciences and Engineering, Nelson Mandela African Institution of Science and Technology, Arusha, Tanzania; 2 Department of Civil and Irrigation Engineering, Mwalimu Julius K. Nyerere University of Agriculture and Technology, Musoma, (Butiama-HQ), Tanzania; 3 Water Infrastructure and Sustainable Energy Futures (WISE-Futures) African Centre of Excellence Road, Tengeru, Arusha, Tanzania; Builders Engineering College, INDIA

## Abstract

The growing demand for sustainable construction materials has increased interest in utilizing agricultural waste as functional additives in fired clay products. However, limited information is available regarding the influence of pyrolysis temperature on the engineering performance, durability, and physicochemical characteristics of biomass-modified burnt red soil bricks. This study investigated the effects of pyrolyzed coffee grounds (PCG) and pyrolyzed tobacco grounds (PTG) produced at 300°C, 350°C, and 500°C and incorporated at replacement levels of 5%, 10%, 15%, and 20% into burnt red soil bricks containing 10% montmorillonite. Mechanical performance was evaluated through compressive, tensile, and flexural strength tests, while durability was assessed using water, chloride, and sulphur penetration measurements. Microstructural and physicochemical characterization was conducted using FTIR, XRD, DSC, Raman spectroscopy, and SEM analyses. Statistical significance was evaluated using analysis of variance (ANOVA) followed by Tukey’s honestly significant difference (HSD) post-hoc test at a 95% confidence level. The results demonstrated that biomass type, pyrolysis temperature, and replacement level significantly influenced both mechanical and durability properties (p < 0.05). Among all investigated mixtures, PCG350−10 exhibited the optimum performance, achieving compressive, tensile, and flexural strengths of 11.8 MPa, 2.8 MPa, and 3.9 MPa, respectively, compared with 8.5 MPa, 1.5 MPa, and 2.2 MPa for the control specimen. Similarly, water, chloride, and sulphur penetration depths decreased from 38 mm, 32 mm, and 35 mm in the control brick to 12 mm, 10 mm, and 11 mm, respectively, representing reductions of approximately 68–69%. Tukey HSD analysis confirmed that PCG350−10 was statistically superior to the control and most alternative formulations. Characterization results revealed progressive thermal transformation of biomass constituents with increasing pyrolysis temperature and indicated that the 350°C condition produced a favorable balance between carbon stabilization, structural integrity, and pore refinement. SEM observations further demonstrated a denser and more homogeneous morphology for the optimum mixtures compared with the control and high-replacement specimens. Overall, the findings demonstrate that incorporating 10% pyrolyzed coffee grounds produced at 350°C significantly enhances the mechanical performance and durability of montmorillonite-modified burnt red soil bricks while providing a sustainable pathway for agricultural waste valorization and resource-efficient construction materials.

## 1. Introduction

The increasing generation of agricultural residues presents significant environmental and waste-management challenges, particularly in developing countries where large quantities of organic by-products are commonly disposed of through open dumping or uncontrolled burning. Converting such residues into value-added construction materials offers a sustainable pathway for reducing environmental burdens while promoting circular resource utilization. Burnt red soil bricks remain among the most widely used masonry materials because of their affordability, local availability, and low manufacturing complexity. However, conventional bricks often exhibit limitations in strength and durability, motivating continued research into the incorporation of supplementary materials capable of improving engineering performance while reducing dependence on virgin resources [1–5].

Numerous studies have investigated the incorporation of agricultural wastes into fired bricks, including rice husk ash, sugarcane bagasse ash, sawdust ash, coffee residues, and other biomass-derived materials. These studies have demonstrated that carefully controlled additions can improve certain physical and mechanical properties while reducing material density and environmental impacts. Nevertheless, excessive replacement levels frequently result in increased porosity, reduced structural integrity, and deterioration of durability performance. Consequently, identifying suitable processing conditions and optimum replacement levels remains a critical challenge for the successful utilization of agricultural waste-derived materials in brick production.

Among emerging biomass-derived additives, pyrolyzed coffee grounds (PCG) and pyrolyzed tobacco grounds (PTG) have attracted increasing attention because pyrolysis transforms unstable organic residues into carbon-rich materials with improved thermal stability and altered physicochemical characteristics. The properties of these pyrolyzed materials are strongly influenced by pyrolysis temperature, which governs carbonization, volatile matter removal, pore development, mineral transformations, and surface chemistry. Previous studies have reported that moderate pyrolysis temperatures can enhance the performance of cementitious and ceramic materials, whereas excessively low or high temperatures may result in less favorable material characteristics. Despite these advances, limited information is available regarding the comparative effects of PCG and PTG produced at different pyrolysis temperatures on the mechanical, durability, and microstructural performance of montmorillonite-modified burnt red soil bricks [6–10].

Montmorillonite has been widely recognized for its high specific surface area, layered structure, and ability to influence particle packing and microstructural development in clay-based systems. Its incorporation into brick matrices may contribute to improved particle interaction and matrix densification when appropriately combined with pyrolyzed biomass-derived materials. However, the combined influence of montmorillonite, pyrolysis temperature, biomass source, and replacement level has not been comprehensively evaluated in burnt red soil bricks. Furthermore, comparative assessments involving both PCG and PTG under identical experimental conditions remain scarce, limiting the understanding of their relative effectiveness and optimum incorporation levels [11–15].

Therefore, this study investigates the mechanical, durability, and microstructural performance of montmorillonite-modified burnt red soil bricks incorporating PCG and PTG produced at 300°C, 350°C, and 500°C and replacement levels ranging from 5% to 20%. Mechanical performance was evaluated through compressive, splitting tensile, and flexural strength tests, while durability was assessed using water, chloride, and sulphur penetration measurements. Microstructural characteristics were examined using FTIR, XRD, SEM, Raman spectroscopy, and DSC analyses. The study aims to identify the optimum biomass source, pyrolysis temperature, and replacement level while providing insight into the relationships between material composition, engineering performance, and microstructural characteristics in sustainable fired-brick systems.

## 2. Materials and methods

### 2.1. Raw materials

The principal raw material used in this investigation was naturally occurring red soil obtained from a local brick-production area in Tanzania. The soil was selected because of its widespread use in traditional fired-brick manufacturing and its availability as a low-cost construction material. Prior to use, the collected soil was air-dried under laboratory conditions to reduce moisture content and facilitate subsequent processing. The dried soil was manually crushed and sieved to remove coarse particles, organic debris, and other impurities that could influence the homogeneity of the mixtures and the firing behavior of the bricks. The processed soil was stored in sealed containers to prevent moisture absorption before specimen preparation.

Montmorillonite (MMT), a layered aluminosilicate clay mineral characterized by high specific surface area and adsorption capacity, was incorporated as a supplementary additive. The material was supplied in powdered form and oven-dried at 105°C for 24 h before use to eliminate residual moisture. The inclusion of montmorillonite was intended to improve particle packing, enhance matrix uniformity, and contribute to microstructural refinement during brick firing.

Spent coffee grounds (SCG) and spent tobacco grounds (STG) were collected from local beverage preparation facilities and tobacco-processing outlets, respectively. These residues were selected because they represent abundant agricultural by-products that are frequently disposed of through landfilling or uncontrolled burning, thereby contributing to environmental pollution. Following collection, the materials were repeatedly washed with distilled water to remove soluble contaminants, residual oils, and other impurities. The cleaned residues were subsequently oven-dried at 105°C for 24 h to achieve a constant mass before pyrolysis. After drying, the materials were stored in airtight containers to prevent moisture uptake and contamination.

The physical and chemical characteristics of the red soil, montmorillonite, and pyrolyzed biomass additives were determined prior to specimen preparation. These properties were used to evaluate their potential influence on packing density, pore development, phase evolution, and mechanical performance of the fired bricks. The measured physical and chemical properties are summarized in Table 1.

**Table 1:** Raw materials used for the production of montmorillonite-modified burnt red soil bricks.

Material	Description	Source Pre-treatment
Red soil	Clayey soil used as the primary matrix material	Air-dried, crushed, and Local source sieved
Montmorillonite (MMT)	Fine aluminosilicate clay mineral used as supplementary additive	Commercial / Oven-dried at 105 °C laboratory grade
Spent coffee grounds (SCG)	Biomass residue from coffee brewing	Washed and oven-dried Local beverage outlets at 105 °C
Spent tobacco grounds (STG)	Biomass residue from tobacco brewing	Washed and oven-dried Local beverage outlets at 105 °C
Water	Mixing and cleaning medium	Tap water Used as received

### 2.2. Pyrolysis of Coffee and Tobacco Grounds

The dried spent coffee grounds and spent tobacco grounds were converted into pyrolyzed coffee grounds (PCG) and pyrolyzed tobacco grounds (PTG) through a controlled pyrolysis process. Pyrolysis was selected because it transforms unstable organic residues into carbon-rich materials with improved thermal stability, modified surface chemistry, and altered pore structures that may influence the performance of fired clay products.

Pyrolysis was conducted in a laboratory muffle furnace operating under limited oxygen conditions. Approximately equal masses of dried SCG and STG were separately placed in heat-resistant containers and introduced into the furnace. Three pyrolysis temperatures, namely 300°C, 350°C, and 500°C, were selected to represent different degrees of carbonization and thermal decomposition. The temperature was increased from ambient conditions to the target temperature at a controlled heating rate of approximately 10°C min^-1^. Upon reaching the target temperature, the materials were maintained under isothermal conditions for 2 h to ensure sufficient thermal conversion.

After completion of the holding period, the furnace was switched off and allowed to cool naturally to room temperature while maintaining limited oxygen exposure. This cooling procedure minimized rapid oxidation of the carbonized products and preserved the structural characteristics developed during pyrolysis. The resulting pyrolyzed materials were removed from the furnace, lightly ground, and sieved to obtain a relatively uniform particle size suitable for brick production as shown in Table 2.

**Table 2:** Pyrolysis conditions of Spent Coffee Grounds (SCG) and Spent Tobacco Grounds (STG).

Additive	Pyrolysis Temperature (°C)	Heating Rate (°C/min)	Holding Time (h)	Atmosphere	Cooling Method
SCG	300	10	2	Limited oxygen	Furnace cooling
SCG	350	10	2		
SCG	500	10	2		
STG	300	10	2		
STG	350	10	2		
STG	500	10	2		

The selected pyrolysis temperatures were chosen based on previous studies indicating that moderate pyrolysis temperatures can improve the physicochemical characteristics of biomass-derived additives, whereas excessively low temperatures may result in incomplete carbonization and excessively high temperatures may lead to the formation of inert ash-rich structures. Detailed pyrolysis parameters including temperature, heating rate, holding time, and atmosphere are presented in Table 3 and 4.

**Table 3:** Physical properties of raw materials and pyrolyzed additives.

Material	Bulk Density (kg/m³)	Particle Size (µm)	Porosity (%)	Specific Gravity	Color
Red Soil	1500	75	35	2.65	Reddish-brown
Montmorillonite	950	20	40	2.40	Light grey
Pyrolyzed Coffee Grounds (PCG)	450	120	65	1.55	Black
Pyrolyzed Tobacco Grounds (PTG)	550	150	55	1.65	Dark brown-black
Fired Brick (Control)	1800	–	25	2.30	Red

**Table 4:** Chemical composition of raw materials and pyrolyzed additives.

Material	SiO_2_ (%)	Al2O_3_ (%)	Fe_2_O_3_ (%)	CaO (%)	MgO (%)	Fixed Carbon (%)	Ash (%)	SiO_2_ + Al_2_O_3_ + Fe_2_O_3_ (%)
Red Soil	60.0	20.0	9.0	2.5	1.5	–	–	89.0
Montmorillonite	52.0	28.0	6.0	1.8	3.5	–	–	86.0
Pyrolyzed Coffee Grounds (PCG)	18.0	14.0	2.0	1.5	0.5	58.0	6.0	34.0
Pyrolyzed Tobacco Grounds (PTG)	10.0	15.0	3.0	2.0	0.7	45.0	24.0	28.0

The combined SiO_2_ + Al_2_O_3_ + Fe_2_O_3_ content is included to facilitate assessment of the aluminosilicate contribution and potential pozzolanic reactivity of the raw materials and pyrolyzed additives.

### 2.3. Physical and chemical properties of materials

The physical properties of the raw materials and pyrolyzed additives were evaluated to assess their potential influence on the engineering performance of the fired bricks. Bulk density, particle size, porosity, specific gravity, and visual appearance were determined for the red soil, montmorillonite, PCG, and PTG. These properties are important because they directly influence particle packing, compaction behavior, shrinkage characteristics, and pore development during drying and firing.

Bulk density measurements were used to evaluate the relative compactness of the materials, while particle size information was employed to assess the ability of the additives to occupy void spaces within the clay matrix. Porosity values provided preliminary indications of the potential contribution of the additives to internal pore development and fluid transport behavior. Specific gravity measurements were used to estimate material density relationships and facilitate mixture design calculations.

Chemical characterization was also conducted to determine the major constituents of the raw materials and additives. The red soil and montmorillonite were characterized in terms of their principal oxide compositions, including SiO_2_, Al_2_O_3_, Fe_2_O_3_, CaO, and MgO. In contrast, the pyrolyzed biomass materials were evaluated based on fixed carbon and ash contents because these parameters strongly influence thermal behavior, firing performance, and the resulting microstructure of the bricks.

The obtained physical and chemical data were subsequently used to interpret mechanical, durability, thermal, and microstructural results. The complete physical and chemical characteristics of the materials.

### 2.4. Mix proportions and brick preparation

Burnt red soil bricks were produced by partially replacing the red soil matrix with pyrolyzed coffee grounds (PCG) or pyrolyzed tobacco grounds (PTG). Replacement levels of 5%, 10%, 15%, and 20% by weight of red soil were investigated to evaluate the influence of additive dosage on mechanical, durability, and microstructural performance. A control mixture containing no pyrolyzed biomass additive was also prepared for comparison purposes.

**Fig 1:**
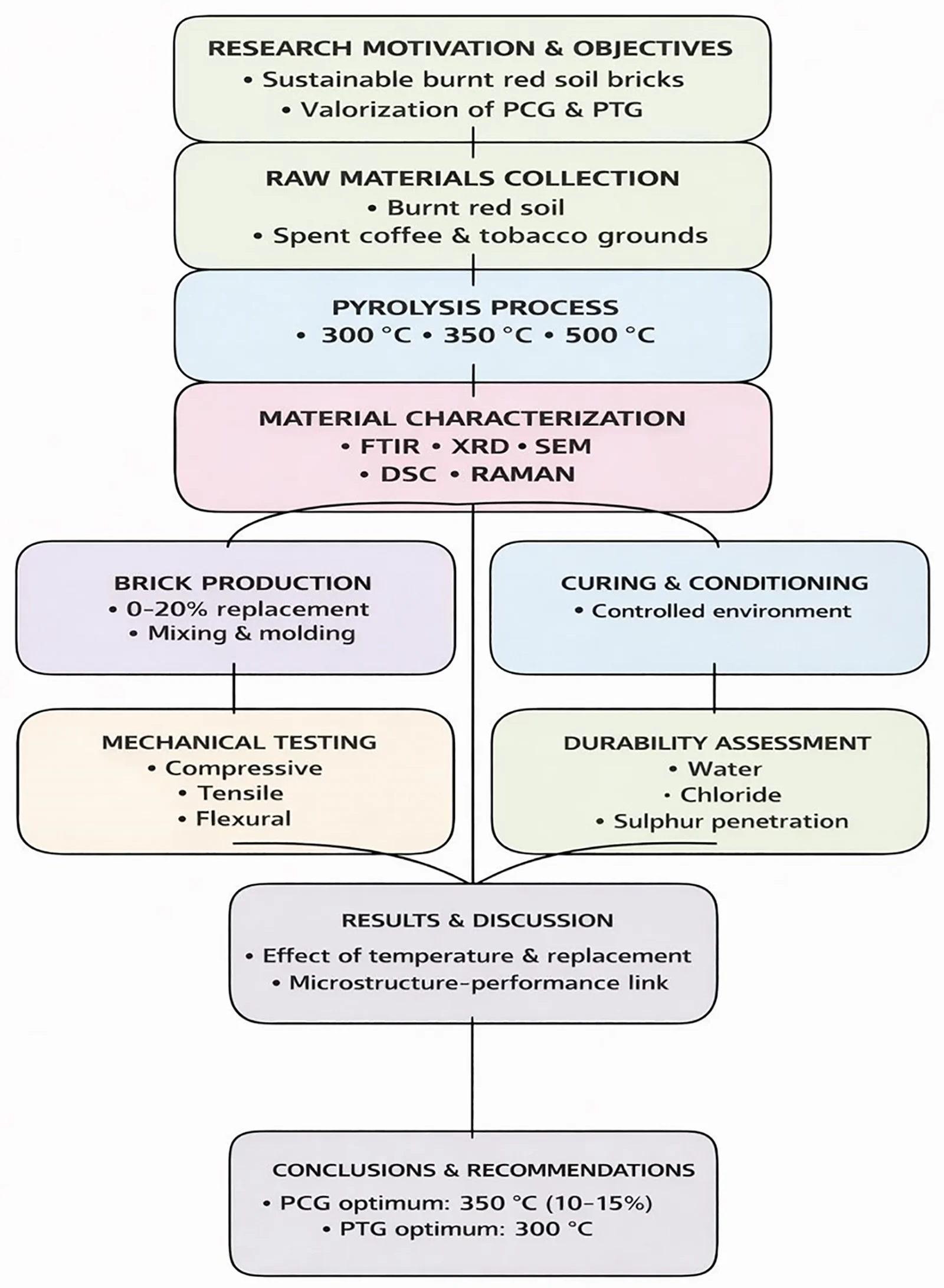
Flowchart of PCG and PTG.

**Fig 2:**
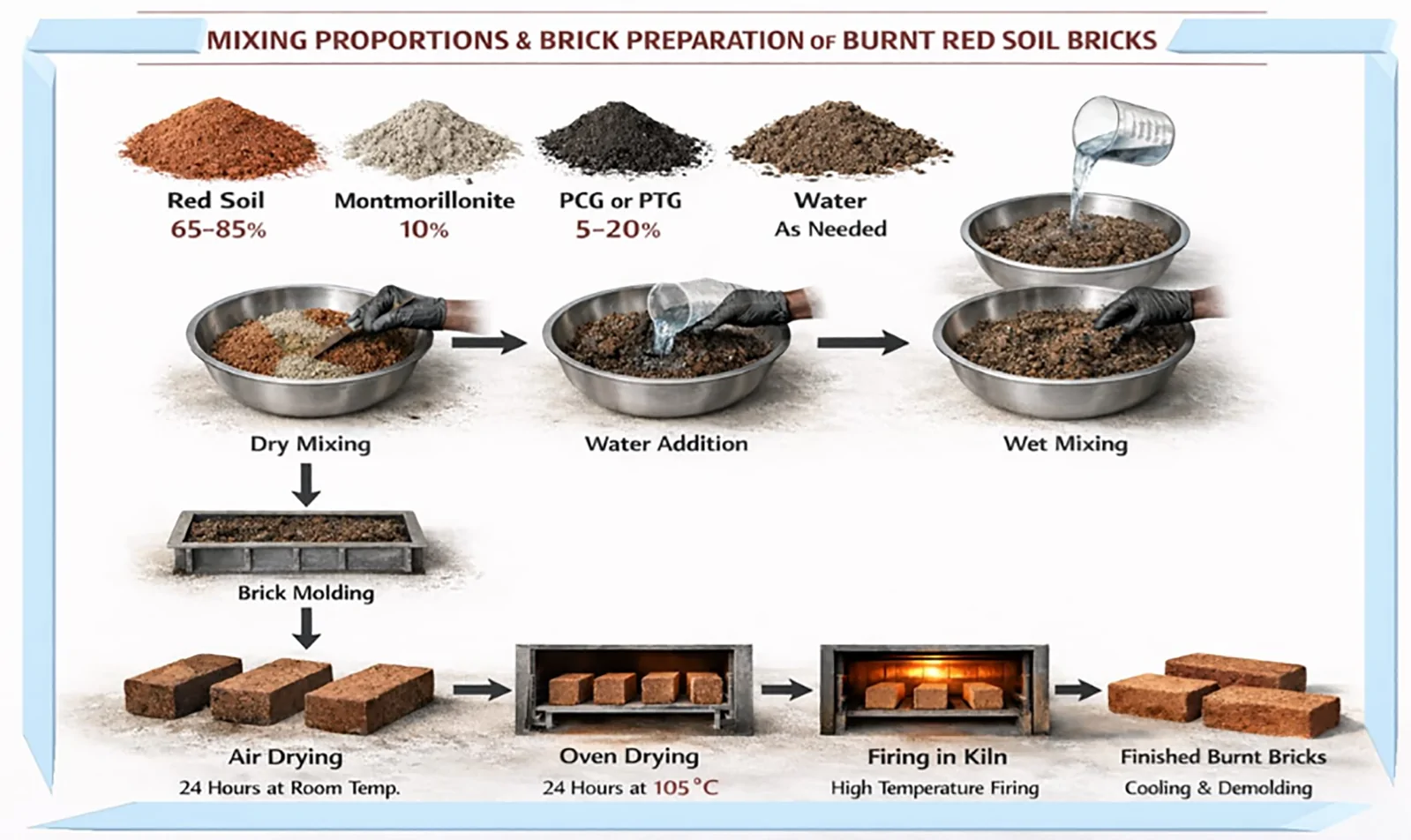
Preparation procedure of PCG and PTG.

In all modified mixtures, montmorillonite was incorporated at a constant dosage of 10% by weight. The inclusion of montmorillonite was intended to enhance particle packing and contribute to matrix development during firing. The complete mixture design and specimen notation adopted in this study are summarized in Table 5 and illustrated schematically in Fig 1 and 2.

**Table 5:** Mix design of montmorillonite–PCG/PTG modified burnt red soil bricks.

Mix ID	Additive type	Pyrolysis temperature (°C)	Montmorillonite content (% by weight)	PCG / PTG replacement (% by weight)
Control	–	–	0	0
MMT–PCG300	PCG	300	10	5, 10, 15, 20
MMT–PCG- 350	PCG	350	10	5, 10, 15, 20
MMT–PCG500	PCG	500	10	5, 10, 15, 20
MMT–PTG300	PTG	300	10	5, 10, 15, 20
MMT–PTG350	PTG	350	10	5, 10, 15, 20
MMT–PTG500	PTG	500	10	5, 10, 15, 20

For each mixture, the required quantities of red soil, montmorillonite, and PCG or PTG were weighed using a calibrated digital balance. The dry constituents were first blended thoroughly to ensure a uniform distribution of particles throughout the mixture. Water was then gradually added while mixing until a workable and homogeneous consistency suitable for molding was obtained.

The fresh mixtures were compacted into standard brick molds and manually leveled to ensure dimensional consistency among specimens. Following molding, the specimens were allowed to air-dry under laboratory conditions for 24 h to minimize rapid moisture loss and reduce the likelihood of drying-induced cracking. Subsequently, the bricks were oven-dried at 105°C for an additional 24 h to remove residual moisture prior to firing.

The dried specimens were fired in a laboratory furnace at 900°C under controlled conditions. This firing temperature was selected because it is representative of temperatures commonly employed in traditional brick manufacturing and is sufficient to promote sintering and phase transformation within the clay matrix. After completion of the firing cycle, the furnace was allowed to cool naturally to room temperature before the specimens were removed.

The fired bricks were visually inspected to identify any visible defects such as cracking, warping, or excessive shrinkage. Specimens exhibiting significant defects were excluded from further testing. The accepted specimens were subsequently subjected to mechanical, durability, thermal, and microstructural evaluations.

### 2.5. Mechanical testing

**Fig 3:**
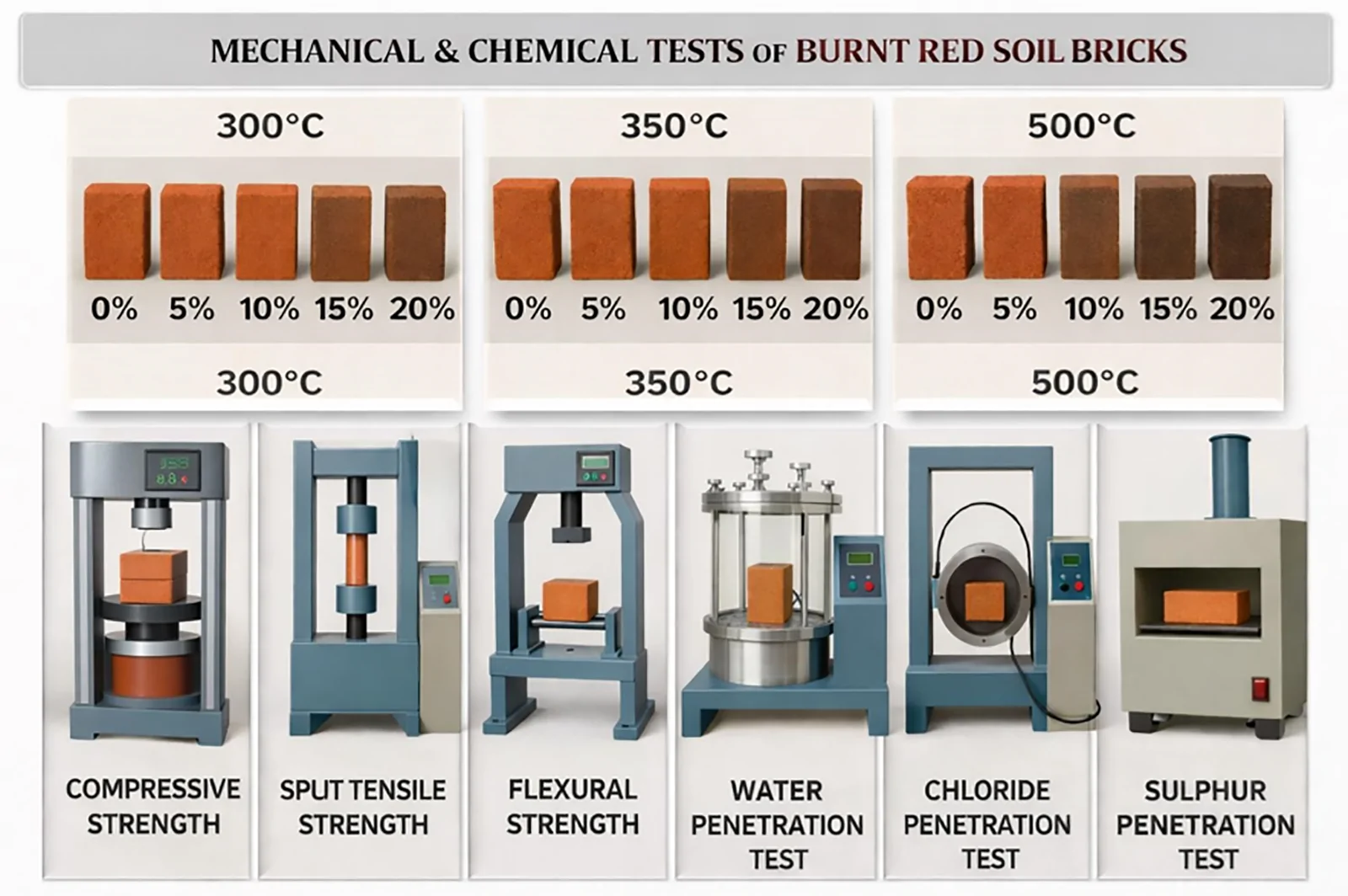
Mechanical & chemical tests of PCG and PTG.

Mechanical performance was evaluated through compressive strength, splitting tensile strength, and flexural strength tests. Compressive strength was determined using uniaxial compression loading, tensile strength was measured using the splitting tensile method, and flexural strength was evaluated using a three-point bending configuration. The testing program is summarized in Table 6 and Fig 3.

**Table 6:** Mechanical and durability testing program for fired bricks.

Test Category	Property Evaluated	Test Method / Exposure Condition	Test Configuration	Measured Parameter
Mechanical	Compressive Strength	Uniaxial Compression Test	Full Brick Loading	Compressive Strength (MPa)
Mechanical	Splitting Tensile Strength	Splitting Tensile Test	Diametral Loading	Tensile Strength (MPa)
Mechanical	Flexural Strength	Three-Point Bending Test	Simply Supported Configuration	Flexural Strength (MPa)
Durability	Water Penetration Resistance	Water Exposure Test	One-Face Exposure	Penetration Depth (mm)
Durability	Chloride Penetration Resistance	Chloride Solution Immersion	Full Immersion	Penetration Depth (mm)
Durability	Sulphate Penetration Resistance	Sulphate Solution Immersion	Full Immersion	Penetration Depth (mm)

Mechanical tests were conducted to evaluate the load-bearing capacity and structural performance of the fired bricks, while durability tests were performed to assess resistance to water, chloride, and sulphate ingress under controlled exposure conditions.

For each mixture and testing age, a minimum of three specimens were tested. The reported results represent the mean values of replicate specimens. Standard deviations (SD), coefficients of variation (COV), and 95% confidence intervals (CI) were subsequently calculated to quantify experimental variability and assess the reliability of measured performance.

### 2.6 Durability testing

Durability performance was assessed through water, chloride, and sulphur penetration tests. Water penetration was evaluated using one-face exposure, while chloride and sulphur penetration were determined through immersion in chloride and sulphate solutions, respectively. After exposure, specimens were split and the maximum penetration depth was measured at several locations and averaged to obtain the final penetration depth.

For each durability condition, a minimum of three replicate specimens were tested. The average penetration depth together with corresponding statistical measures was used for subsequent analysis. The testing program and measured parameters are summarized in Table 6.

### 2.7. FTIR analysis

Fourier Transform Infrared Spectroscopy (FTIR) was performed using an ATR-FTIR spectrometer over the wavenumber range of 4000–500 cm^-1^. The analysis was conducted to identify functional groups and chemical bonding characteristics present in PCG and PTG produced at different pyrolysis temperatures. The obtained spectra were used to evaluate changes in surface chemistry associated with pyrolysis.

### 2.8. XRD analysis

X-ray diffraction (XRD) analysis was conducted using Cu-Kα radiation over a 2θ range of 5°–70°. The technique was employed to identify crystalline phases and evaluate structural changes associated with pyrolysis and brick firing. Phase identification was performed by comparing diffraction peaks with standard reference patterns.

### 2.9. SEM analysis

Scanning Electron Microscopy (SEM) was used to examine the morphology, pore structure, and microstructural characteristics of selected brick specimens. Particular attention was given to the distribution of pyrolyzed additives, matrix continuity, and the presence of pores and microcracks that could influence mechanical and durability performance.

### 2.10. DSC analysis

Differential Scanning Calorimetry (DSC) was employed to evaluate the thermal behavior and stability of PCG and PTG. Samples were heated from ambient temperature to approximately 800°C under controlled conditions, and thermal events were recorded to assess decomposition characteristics and thermal stability. The DSC testing conditions are summarized in Table 7.

**Table 7:** Characterization and statistical analysis techniques.

Technique	Instrument / Method	Measurement Conditions	Purpose
FTIR	ATR–FTIR spectrometer	4000–500 cm^-1^	Identification of functional groups and chemical bonding in PCG and PTG
XRD	X-ray diffractometer (Cu–Kα radiation)	2θ range: 5°–70°	Phase identification and crystallinity analysis
SEM	Scanning Electron Microscope	High-vacuum mode	Microstructural observation, pore structure, and interfacial bonding
DSC	Differential Scanning Calorimeter	Room temperature to 800 °C	Thermal behavior and stability analysis of PCG and PTG
Raman	Micro-Raman spectrometer	800–2000 cm^-1^	Carbon structural ordering and defect characterization

### 2.11. Raman spectra analysis

Raman spectroscopy was performed over the range of 800–2000 cm^-1^ to evaluate the structural characteristics of carbon present in PCG and PTG. Particular emphasis was placed on the D and G bands associated with disordered and graphitic carbon structures. The obtained spectra were used to assess the influence of pyrolysis temperature on carbon ordering.

### 2.12. Data analysis

All experimental data were analyzed using the mean values obtained from replicate specimens. Mechanical, durability, and microstructural results were compared to evaluate the effects of biomass source, pyrolysis temperature, and replacement level. Variability measures including standard deviation, coefficient of variation, and confidence intervals were also determined.

### 2.13. Statistical analysis (ANOVA)

Statistical analysis was performed to evaluate the significance of pyrolysis temperature and replacement level on mechanical and durability performance. Two-way analysis of variance (ANOVA) was conducted separately for PCG- and PTG-modified bricks to determine the individual and interaction effects of pyrolysis temperature and replacement percentage. Statistical significance was evaluated at a confidence level of 95% (α = 0.05), and p-values less than 0.05 were considered significant. When significant differences were detected, Tukey’s Honestly Significant Difference (HSD) post-hoc test was performed to identify pairwise differences among mixture combinations. The results are reported in terms of sums of squares (SS), degrees of freedom (df), mean squares (MS), F-values, p-values, and multiple-comparison statistics.

## 3. Results and discussion

### 3.1. Mechanical properties

#### 3.1.1. Compressive strength.

**Fig 4:**
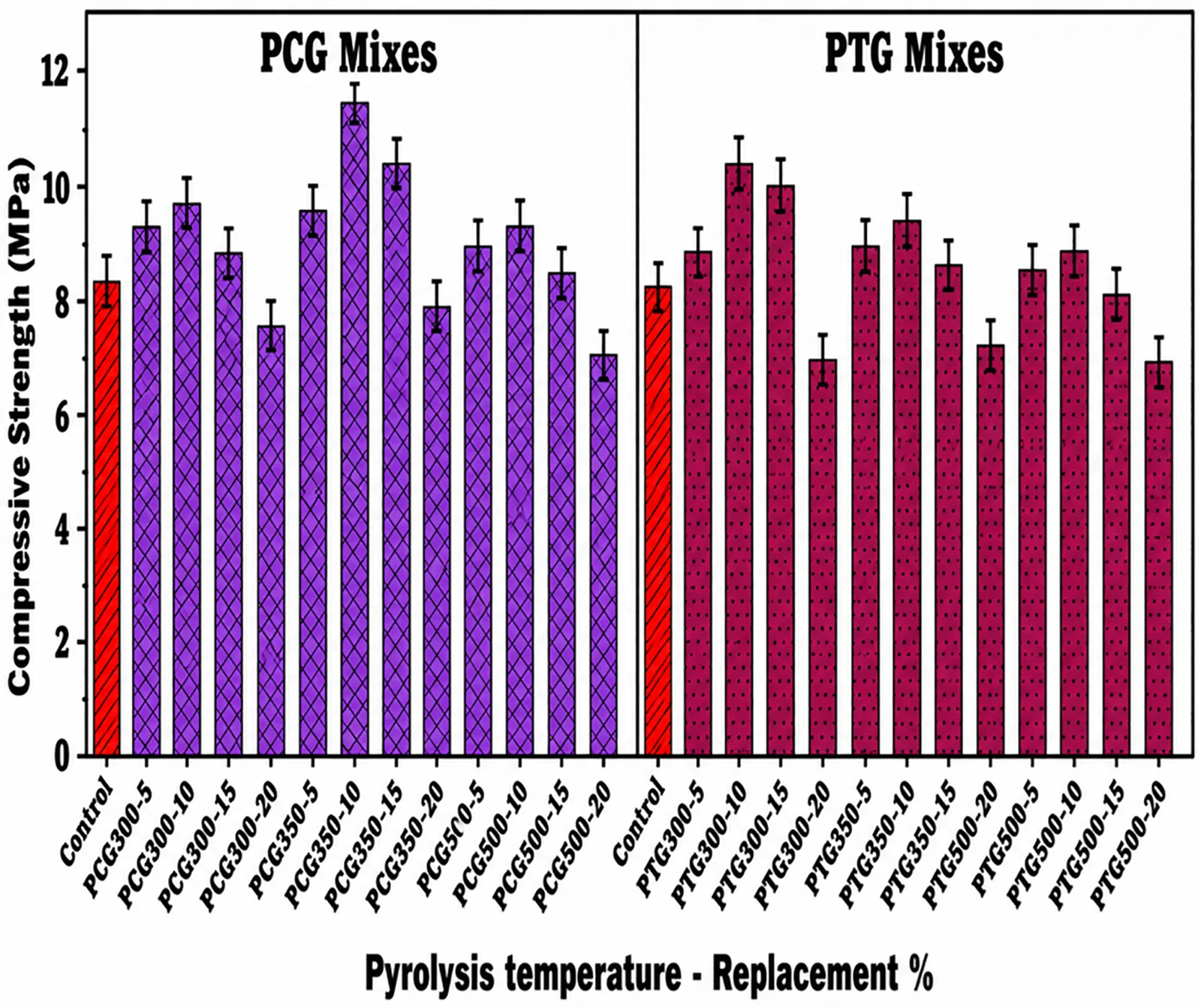
Compressive strength of PCG and PTG.

The compressive strength results of burnt red soil bricks incorporating pyrolyzed coffee grounds (PCG) and pyrolyzed tobacco grounds (PTG) at different pyrolysis temperatures and replacement levels are presented in Fig 4. Compressive strength is one of the most important indicators of brick quality because it reflects the ability of the material to withstand external loads and resist crack initiation under compressive stress. The results demonstrate that both pyrolysis temperature and replacement percentage significantly influenced compressive strength, indicating that the thermal treatment of biomass plays a critical role in determining the engineering performance of the resulting bricks.

Compared with the control specimen (8.5 MPa), most modified mixtures exhibited improved compressive strength. For the PCG series, strength increased from 9.3 MPa at PCG300−5 to 11.8 MPa at PCG350−10, representing an improvement of approximately 38.8% over the control. Similarly, PTG-modified bricks generally exhibited higher compressive strengths than the control, with PTG300−10 achieving the highest value of 10.6 MPa. However, increasing the replacement level beyond the optimum percentage resulted in gradual strength reduction. For example, PCG350−15 and PCG350−20 recorded compressive strengths of 10.6 MPa and 7.9 MPa, respectively, while PTG500−20 exhibited the lowest strength of 6.9 MPa.

The observed improvement at moderate replacement levels can be attributed to several complementary mechanisms. First, pyrolysis removes volatile organic compounds and converts the biomass into a more stable carbonaceous material. This process reduces dimensional instability and promotes compatibility with the clay matrix during brick firing. Second, the fine particles generated during pyrolysis may act as microfillers that occupy void spaces between clay particles, thereby improving particle packing density and reducing pore connectivity. Improved packing reduces stress concentration sites and enhances the efficiency of load transfer throughout the matrix. Third, the presence of mineral-rich ash components may contribute additional silica- and alumina-bearing phases that become integrated into the ceramic structure during firing.

The superior performance of PCG350−10 suggests that pyrolysis at 350°C produced the most favorable balance between carbonization and functional-group preservation. At temperatures below 350°C, incomplete decomposition of cellulose, hemicellulose, and lignin may leave unstable organic residues within the biomass. During firing, these residues can decompose further, generating gases and increasing pore formation within the brick matrix. Such defects reduce density and negatively affect compressive strength. Conversely, at temperatures above 350°C, excessive carbonization may result in structural shrinkage, particle fragmentation, and reduced surface activity of the biomass-derived additive. These effects can diminish the beneficial filler action and reduce the effectiveness of matrix reinforcement.

Another important observation is the influence of replacement percentage. While additions of 5–15% generally improved compressive strength, replacement levels of 20% resulted in noticeable reductions. This behavior suggests that excessive substitution disrupts the continuity of the clay matrix and introduces a larger volume of non-plastic material into the mixture. As the amount of pyrolyzed biomass increases, the available clay fraction decreases, reducing the ability of the matrix to densify effectively during firing. Consequently, excessive replacement levels may increase porosity and decrease the overall structural integrity of the bricks.

The superior performance of PCG compared with PTG may also be related to differences in biomass composition. Coffee grounds generally contain higher proportions of carbonaceous material and produce a more favorable porous carbon structure after pyrolysis. In contrast, tobacco-derived residues may contain greater amounts of inorganic impurities and ash-forming components, which can alter firing behavior and reduce the efficiency of matrix densification. These differences likely contributed to the consistently higher compressive strengths observed for PCG mixtures.

Overall, the results demonstrate that both biomass type and pyrolysis temperature significantly affected compressive strength. Among all investigated mixtures, PCG350−10 achieved the highest compressive strength (11.8 MPa), indicating that a pyrolysis temperature of 350°C combined with a replacement level of 10% produced the most favorable structural condition for load-bearing applications.

#### 3.1.2 Tensile strength.

The tensile strength results followed trends similar to those observed for compressive strength. Tensile strength is particularly important because brittle ceramic materials such as fired bricks typically fail under tensile stresses generated by shrinkage, bending, or external loading. Therefore, improvements in tensile strength indicate enhanced resistance to crack initiation and propagation within the brick matrix.

The control specimen exhibited a tensile strength of 1.5 MPa, whereas all optimum PCG and PTG mixtures achieved considerably higher values. For the PCG series, tensile strength increased from 1.9 MPa at PCG300−5 to a maximum value of 2.8 MPa at PCG350−10, representing an increase of approximately 86.7% relative to the control. Similarly, PTG300−10 achieved a tensile strength of 2.4 MPa. However, increasing the replacement percentage to 20% resulted in substantial reductions, with PCG350−20 and PTG500−20 recording values of 1.8 MPa and 1.7 MPa, respectively.

**Fig 5:**
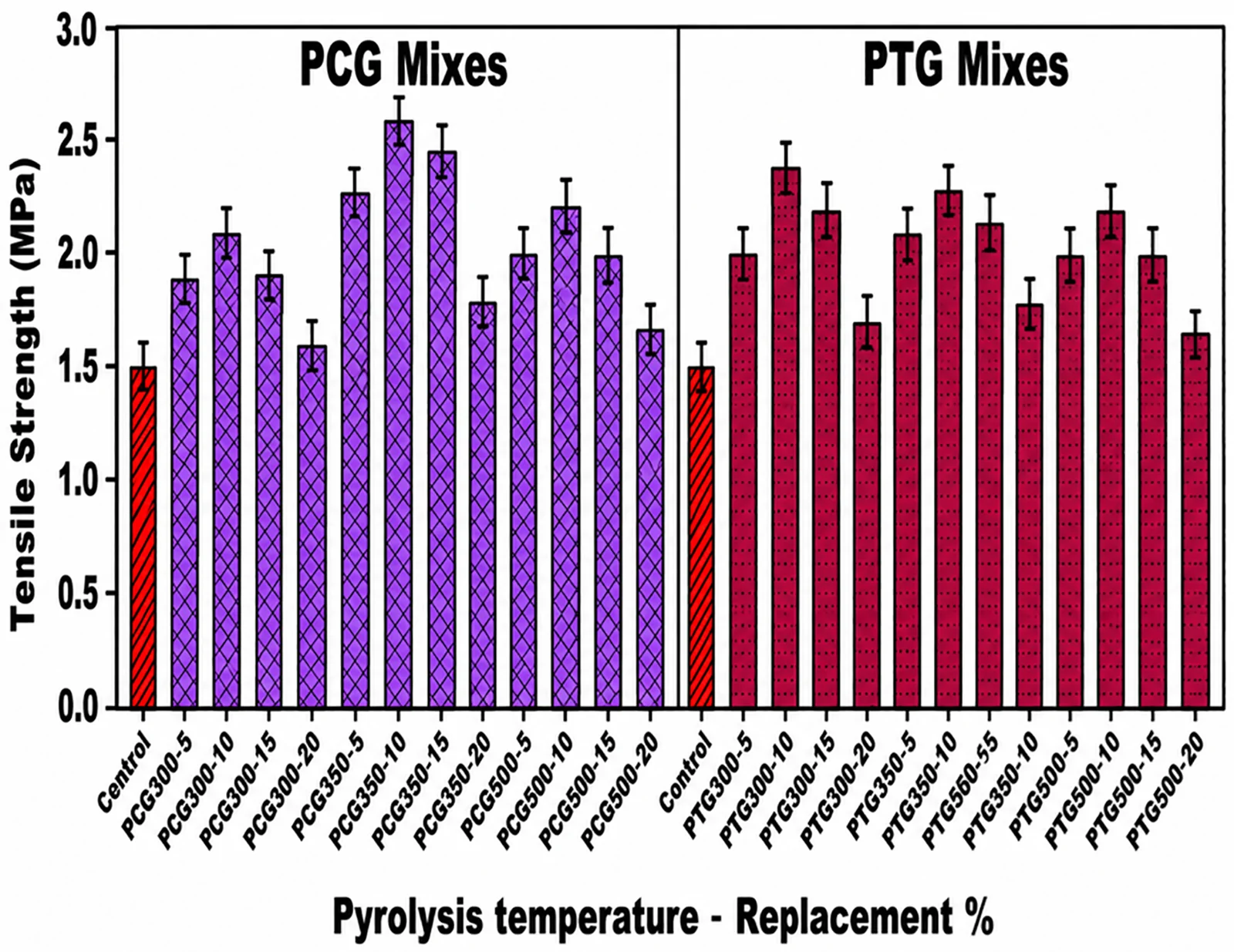
Tensile strength of PCG and PTG.

The increase in tensile strength at moderate replacement levels can be explained by improved microstructural uniformity and reduced defect concentration within the fired matrix. Cracks generally initiate at pores, weak interfaces, and microstructural discontinuities. The incorporation of appropriately pyrolyzed biomass particles may reduce the size and distribution of such defects by improving particle packing and promoting a more homogeneous internal structure. As a result, higher stresses are required to initiate and propagate cracks through the material [4–8,16–18] as shown in Fig 5.

Furthermore, pyrolysis at 350°C appears to generate carbonaceous particles with sufficient structural integrity to contribute to crack-arrest mechanisms. When microcracks encounter well-dispersed carbonaceous inclusions, crack propagation may be partially deflected or delayed. Although the contribution of such mechanisms is difficult to quantify directly, the consistently higher tensile strengths observed for the 350°C mixtures suggest that the resulting microstructure was more resistant to tensile failure.

The reduction in tensile strength at higher replacement levels may be attributed to increased pore formation and reduced matrix continuity. Excessive incorporation of biomass-derived particles introduces additional interfaces within the ceramic structure. These interfaces can act as weak zones under tensile loading and facilitate crack propagation. In addition, higher biomass contents may generate greater amounts of volatile gases during firing, increasing the likelihood of pore development and reducing tensile resistance [6–10].

The influence of pyrolysis temperature was also evident. Specimens produced from biomass pyrolyzed at 300°C generally exhibited lower tensile strengths because of incomplete thermal decomposition and residual organic matter. Conversely, specimens produced from biomass pyrolyzed at 500°C showed evidence of excessive carbonization, which may reduce the beneficial interaction between the additive and the clay matrix. Therefore, the optimum tensile performance observed at 350°C reflects a balance between thermal stabilization and preservation of structurally beneficial carbonaceous features [4–8,16–18].

Overall, PCG350−10 exhibited the highest tensile strength (2.8 MPa), confirming that this combination of pyrolysis temperature and replacement level provided the most favorable conditions for resisting crack initiation and propagation within the brick matrix.

#### 3.1.3. Flexural strength.

Flexural strength provides an assessment of the ability of bricks to resist bending stresses and is often considered a more sensitive indicator of microstructural quality than compressive strength. Under flexural loading, tensile and compressive stresses are generated simultaneously, making flexural strength highly dependent on defect distribution, matrix continuity, and crack resistance.

The control specimen exhibited a flexural strength of 2.2 MPa. All optimum PCG and PTG mixtures exceeded this value, demonstrating the beneficial influence of pyrolyzed biomass incorporation. Among the PCG mixtures, flexural strength increased progressively to a maximum value of 3.9 MPa for PCG350−10, representing an improvement of approximately 77.3% relative to the control. Similarly, PTG300−10 achieved a flexural strength of 3.4 MPa. However, mixtures containing 20% replacement generally exhibited lower flexural strengths, indicating that excessive biomass incorporation adversely affected structural performance.

The enhancement in flexural strength can be attributed to improved matrix densification and reduced pore connectivity. During bending, stress concentrations develop around pores and microcracks. A denser and more homogeneous microstructure distributes stresses more uniformly and delays crack initiation. The carbonaceous particles generated through pyrolysis may also contribute to bridging microstructural discontinuities and reducing local stress concentrations.

The exceptional performance of PCG350−10 suggests that pyrolysis at 350°C generated a material with favorable morphological and physicochemical characteristics. At this temperature, sufficient removal of volatile matter occurred to minimize defect formation, while the remaining carbonaceous structure retained enough integrity to contribute positively to matrix organization. Consequently, the resulting brick exhibited superior resistance to bending-induced crack propagation.

The decline in flexural strength observed at 20% replacement levels is consistent with the increased likelihood of pore formation and matrix disruption. Excessive biomass addition reduces the proportion of cohesive clay material and increases the number of interfaces within the matrix. These interfaces may act as preferential crack paths under bending loads, thereby reducing flexural capacity.

**Fig 6:**
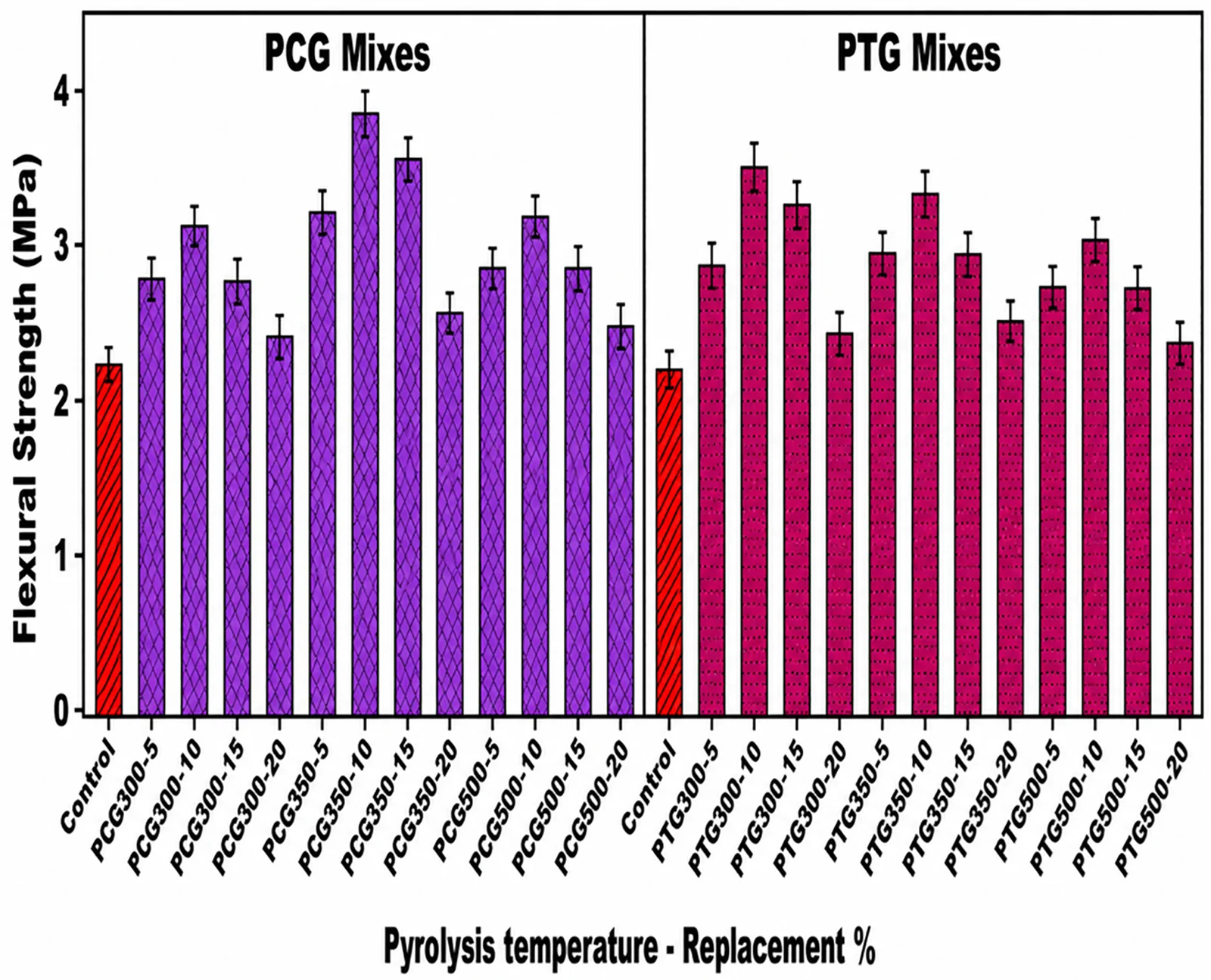
Flexural strength of PCG and PTG.

Comparison of PCG and PTG mixtures revealed that PCG generally produced superior flexural performance. This observation may be related to differences in carbon yield, ash content, and particle morphology between the two biomass sources. The more favorable carbonaceous structure generated from coffee grounds appears to have contributed to greater matrix uniformity and improved stress transfer within the fired bricks as presented in Fig 6.

Among all investigated mixtures, PCG350−10 consistently exhibited the highest compressive strength (11.8 MPa), tensile strength (2.8 MPa), and flexural strength (3.9 MPa). The consistency of this trend across all mechanical tests strongly indicates that pyrolysis at 350°C and a replacement level of 10% produced the most favorable balance between biomass transformation, matrix densification, and structural integrity. The microstructural and physicochemical mechanisms responsible for this behavior are further explored in the subsequent characterization sections.

#### 3.1.4. Water penetration.

The control specimen exhibited the highest water penetration depth (38 mm), indicating a relatively open and interconnected pore network that facilitated capillary water transport. In contrast, all optimum PCG and PTG mixtures exhibited lower penetration depths, demonstrating enhanced resistance to moisture ingress. The lowest value was recorded for PCG350−10 (12 mm), representing a 68.4% reduction relative to the control. Similarly, PTG300−10 (18 mm) reduced water penetration by 52.6% compared with the control.

**Fig 7:**
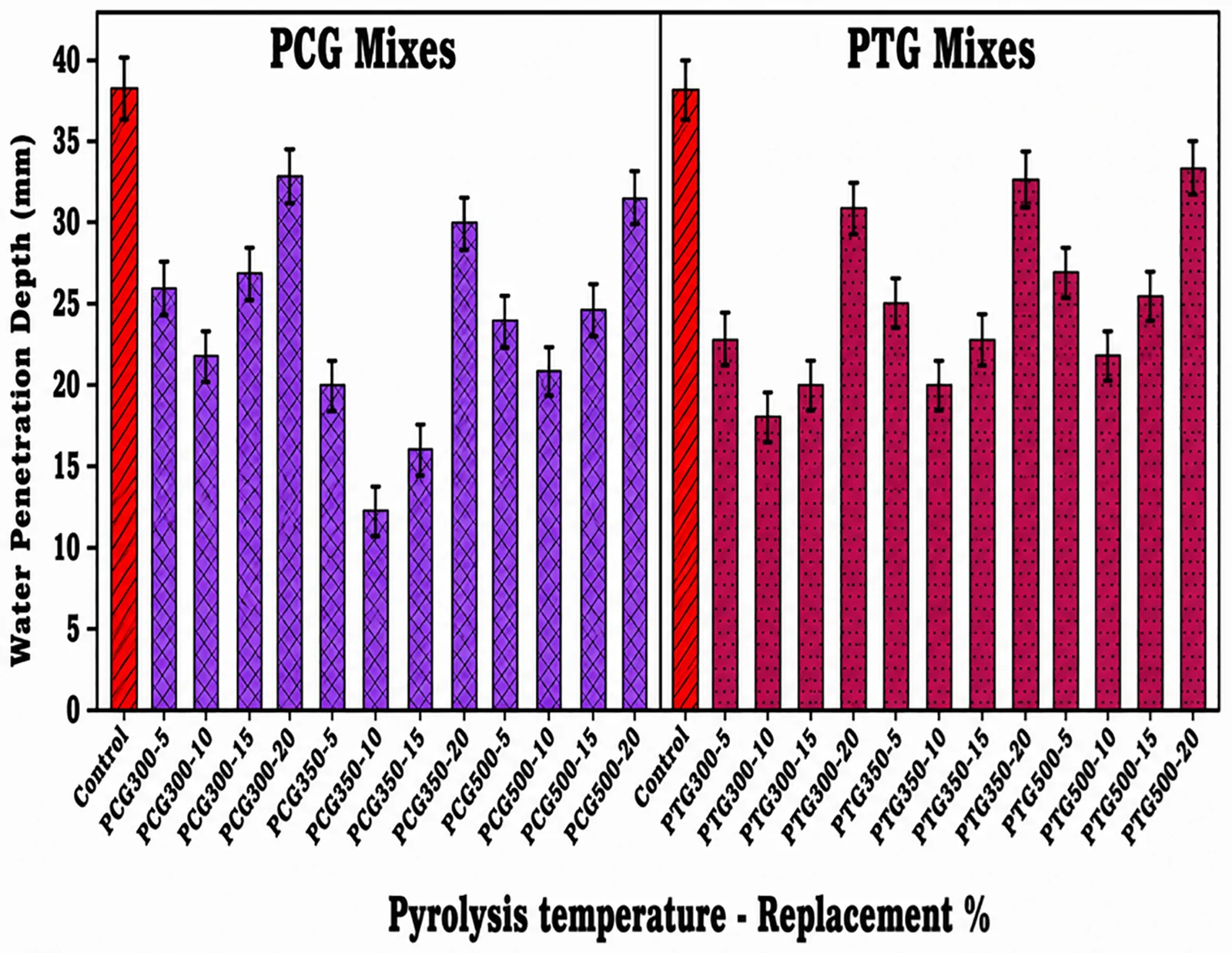
Water penetration of PCG and PTG.

The substantial reduction in penetration depth indicates that the modified bricks developed a denser internal structure than the control brick. While water could readily migrate through the interconnected pores of the control specimen, the incorporation of pyrolyzed biomass reduced pore continuity and increased the tortuosity of transport pathways. Consequently, moisture movement became more restricted, leading to lower penetration depths. The superior performance of PCG350−10 suggests that this mixture achieved the highest degree of pore refinement and matrix densification among all investigated specimens as illustrated in Fig 7.

#### 3.1.5. Chloride penetration.

The control brick recorded a chloride penetration depth of 32 mm, indicating relatively low resistance to ionic transport. All modified mixtures performed better than the control, with the greatest improvement observed for PCG350−10, which exhibited a penetration depth of 10 mm. This corresponds to a 68.8% reduction compared with the control. Likewise, PTG300−10 reduced chloride penetration to 15 mm, representing a 53.1% improvement.

**Fig 8:**
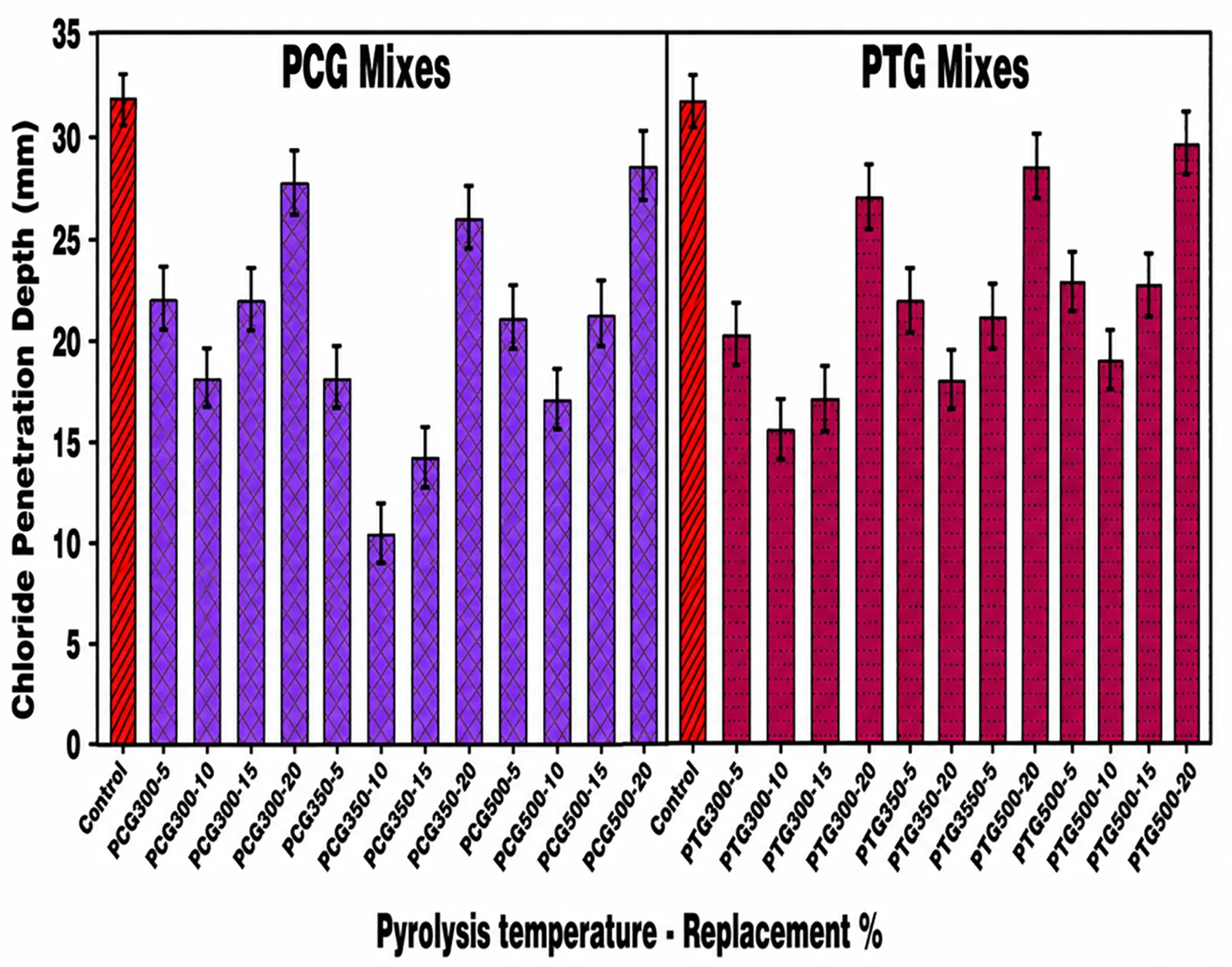
Chloride penetration of PCG and PTG.

Compared with the control, the reduced chloride penetration depths indicate that the modified bricks possessed fewer continuous transport pathways for ionic movement. Chloride ions migrate primarily through water-filled pores; therefore, the lower penetration depths suggest a reduction in accessible porosity and an increase in diffusion resistance. The outstanding performance of PCG350−10 indicates that this mixture provided the most effective barrier against chloride ingress, thereby enhancing long-term durability as shown in Fig 8.

#### 3.1.6. Sulphur penetration.

The control specimen exhibited the highest sulphur penetration depth (35 mm), reflecting its greater susceptibility to aggressive sulphate-bearing environments. In contrast, all optimum modified bricks demonstrated improved resistance. The minimum penetration depth was recorded for PCG350−10 (11 mm), representing a 68.6% reduction compared with the control. Similarly, PTG300−10 (16 mm) achieved a 54.3% reduction relative to the control specimen.

**Fig 9:**
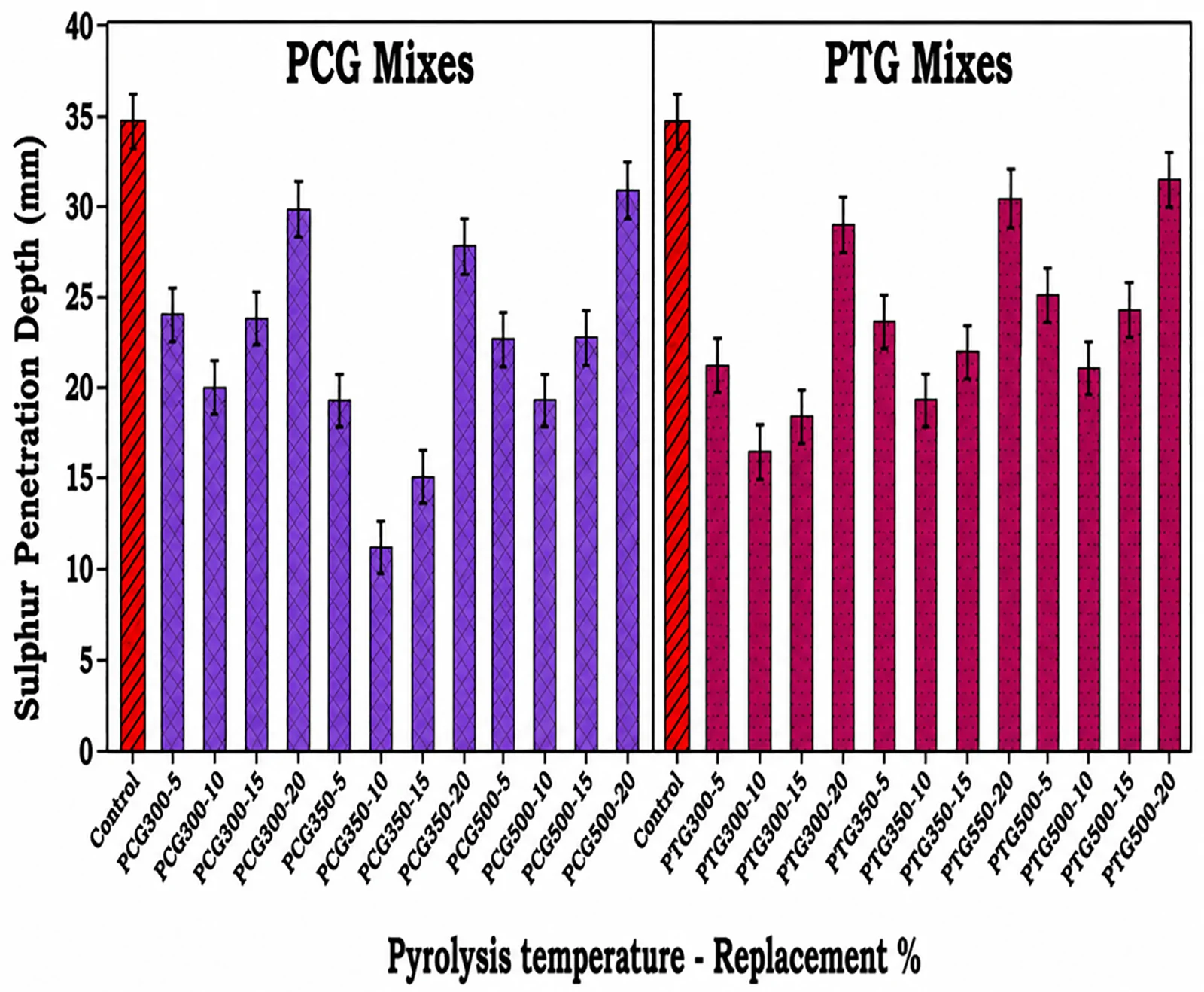
Sulphur penetration of PCG and PTG.

The improved sulphur resistance of the modified bricks indicates a significant reduction in permeability compared with the control. Whereas aggressive solutions could penetrate deeply into the control brick through interconnected capillary pores, the modified mixtures restricted fluid transport by reducing pore connectivity and increasing transport resistance. The consistently lower penetration depths observed for PCG350−10 across all durability tests confirm that this mixture developed the most compact and least permeable internal structure as shown in Fig 9.

**Overall durability comparison with the control:** Compared with the control brick, PCG350−10 exhibited the greatest overall durability enhancement, reducing water penetration from 38 mm to 12 mm (68.4%), chloride penetration from 32 mm to 10 mm (68.8%), and sulphur penetration from 35 mm to 11 mm (68.6%). These reductions were accompanied by simultaneous improvements in compressive strength (8.5 MPa to 11.8 MPa; + 38.8%), tensile strength (1.5 MPa to 2.8 MPa; + 86.7%), and flexural strength (2.2 MPa to 3.9 MPa; + 77.3%). The simultaneous enhancement of both mechanical and durability properties suggests that the optimum mixture achieved a more compact, less permeable, and structurally efficient matrix than the control specimen. This finding is further supported by the microstructural and physicochemical analyses presented in Section 3.3.

### 3.2. Microstructural and physicochemical characterization

#### 3.2.1. FTIR analysis.

**Fig 10:**
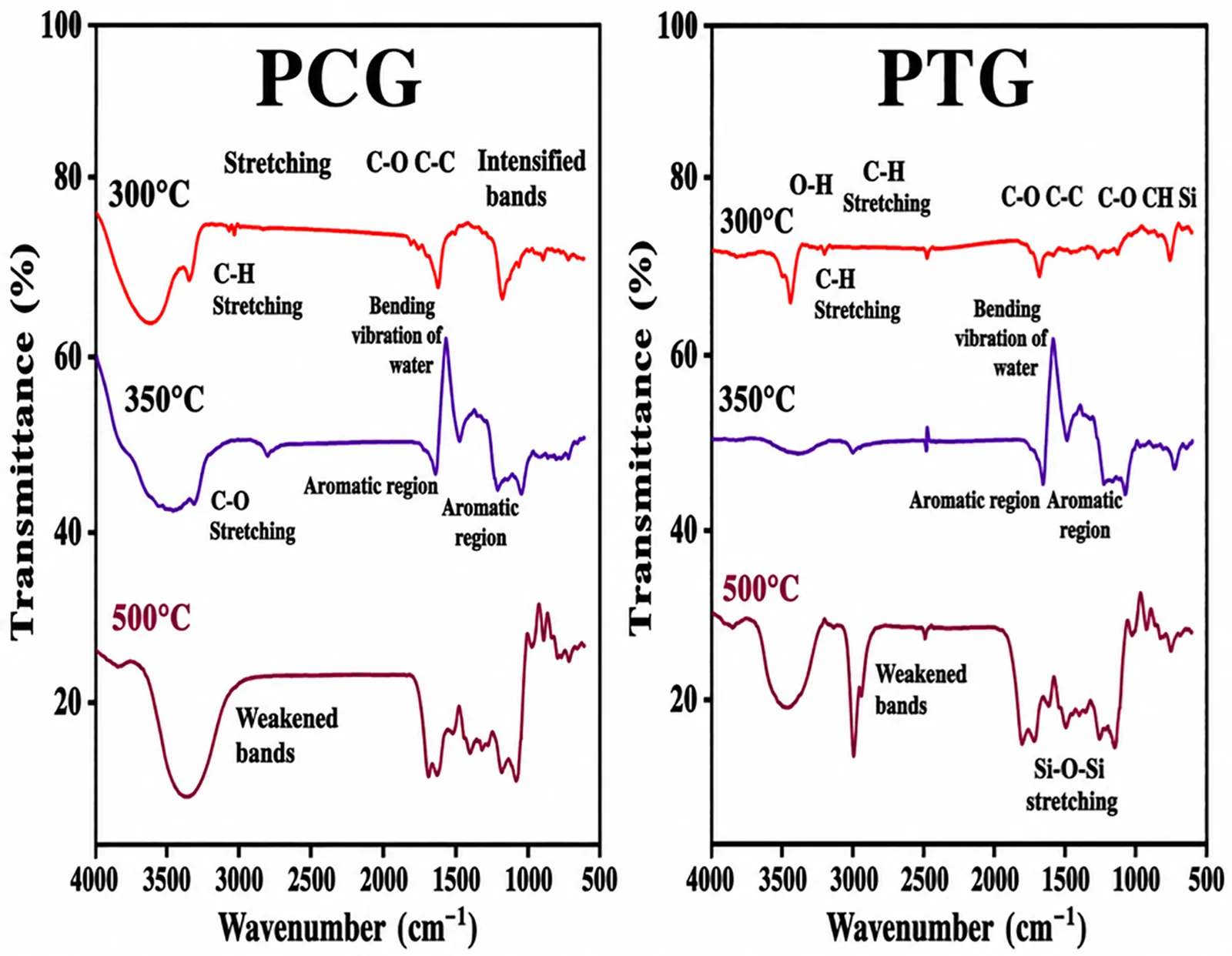
FTIR analysis of PCG and PTG.

The FTIR spectra of pyrolyzed coffee grounds (PCG) and pyrolyzed tobacco grounds (PTG) produced at 300°C, 350°C, and 500°C are presented in Fig 10. The spectra exhibited characteristic absorption bands associated with hydroxyl groups, aliphatic carbon structures, aromatic compounds, oxygen-containing functionalities, and mineral-related phases. Progressive changes in peak intensity and definition were observed with increasing pyrolysis temperature, indicating substantial physicochemical transformation of the biomass materials during thermal treatment.

**PCG and PTG at 300°C:** The spectra of PCG300 and PTG300 exhibited a broad absorption band within the range of approximately 3600–3200 cm^-1^, corresponding to O–H stretching vibrations associated with hydroxyl groups and adsorbed moisture. The pronounced intensity of this band indicates the retention of moisture-containing compounds and incompletely decomposed biomass constituents following low-temperature pyrolysis. Distinct absorption peaks were also observed near 2920–2850 cm^-1^, corresponding to aliphatic C–H stretching vibrations. The presence of these peaks suggests that cellulose-, hemicellulose-, and lignin-derived structures were not completely decomposed at 300°C.

Additional absorption features were observed near 1723 cm^-1^ and 1640 cm^-1^, which were attributed to C = O stretching vibrations and H–O–H bending or aromatic C = C vibrations, respectively. The persistence of these bands further confirms the presence of oxygen-containing organic structures. The region between 1100 and 980 cm^-1^ exhibited absorption bands associated with C–O, Si–O–Si, and Si–O–Al stretching vibrations, indicating contributions from both organic and mineral phases. These observations suggest that pyrolysis at 300°C resulted in partial carbonization while retaining a substantial proportion of reactive organic functionalities.

From an engineering perspective, the incomplete thermal transformation observed at 300°C corresponded with moderate mechanical and durability performance. For example, PCG300−10 achieved a compressive strength of approximately 9.8 MPa, tensile strength of 2.1 MPa, and flexural strength of 3.1 MPa, together with water, chloride, and sulphur penetration depths of approximately 24 mm, 18 mm, and 23 mm, respectively. These values were lower than those obtained for the corresponding 350°C specimens.

**PCG and PTG at 350°C:** The FTIR spectra of PCG350 and PTG350 showed noticeable reductions in the O–H stretching band and the aliphatic C–H stretching peaks relative to the corresponding 300°C specimens. These reductions indicate progressive dehydration and decomposition of unstable organic compounds during pyrolysis. However, unlike the 500°C specimens, distinct absorption bands remained visible within the aromatic and oxygen-containing regions.

The absorption bands near 1723 cm^-1^, 1640 cm^-1^, and 1522 cm^-1^ became more defined, indicating the presence of stabilized aromatic and partially carbonized structures. Similarly, the bands within the range of 1100–980 cm^-1^ remained prominent, suggesting the coexistence of carbonaceous and mineral-related phases. The carbonate-related bands near 875 cm^-1^ and 712 cm^-1^ were also identifiable, indicating the presence of thermally stable inorganic constituents.

These spectral characteristics suggest that pyrolysis at 350°C promoted substantial carbonization while preserving a portion of the oxygen-containing and aromatic functionalities. Such a balance may provide improved physicochemical stability without complete loss of surface activity. Among all investigated mixtures, PCG350−10 exhibited the highest compressive strength (11.8 MPa), tensile strength (2.8 MPa), and flexural strength (3.9 MPa), together with the lowest water (12 mm), chloride (10 mm), and sulphur (11 mm) penetration depths. Although FTIR analysis does not directly measure strength or durability, the functional-group distribution observed at 350°C is consistent with the superior engineering performance obtained for the corresponding brick specimens as shown in Fig 10 and Table 8.

**Table 8:** Summary of FTIR analysis of PCG and PTG.

Material	Temperature (°C)	Main Observation	Interpretation
PCG	300	Strong O–H, C–H, and C–O bands	Incomplete carbonization; residual organic matter present
PCG	350	Reduced O–H and C–H bands; enhanced aromatic region	Optimum balance between carbonization and functional group retention
PCG	500	Weakened functional-group bands	Extensive carbonization and loss of reactive groups
PTG	300	Distinct O–H, C–H, and C–O–C bands	Partial carbonization with retained organics
PTG	350	Strong aromatic region with reduced O–H and C–H bands	Improved carbon ordering and thermal stability
PTG	500	Weak functional-group bands; pronounced Si–O–Si peak	Advanced carbonization and mineral enrichment

Overall Finding

Increasing pyrolysis temperature reduced O–H and C–H functional groups and enhanced aromatic carbon formation. The 350°C biochars (especially PCG350) exhibited the most favorable physicochemical characteristics, explaining their superior mechanical strength and durability performance.

**PCG and PTG at 500°C:** The spectra of PCG500 and PTG500 exhibited the greatest degree of thermal transformation. The O–H stretching band became substantially weaker, while the aliphatic C–H stretching peaks near 2920–2850 cm^-1^were significantly diminished. Likewise, the intensity of the oxygen-containing bands near 1723 cm^-1^ and within the 1100–980 cm^-1^ region decreased considerably, indicating extensive decomposition of organic functional groups.

In contrast, mineral-related bands became more pronounced, particularly within the silicate region below approximately 1100 cm^-1^. This behavior suggests concentration of inorganic and ash-related components following removal of volatile organic matter. The reduced intensity of oxygen-containing functional groups indicates that pyrolysis at 500°C produced a more carbonized and mineral-rich material with lower chemical functionality.

The extensive thermal decomposition observed at 500°C corresponded with lower engineering performance than that observed at 350°C. For example, PCG500−10 achieved a compressive strength of approximately 9.4 MPa, tensile strength of 2.2 MPa, and flexural strength of 3.2 MPa, together with water, chloride, and sulphur penetration depths of approximately 21 mm, 17 mm, and 19 mm, respectively. These results suggest that excessive carbonization may reduce the beneficial contribution of the pyrolyzed material to the fired-brick matrix.

**Comparative Interpretation:** Comparison of the spectra demonstrates that increasing pyrolysis temperature progressively reduced hydroxyl, aliphatic, and oxygen-containing functional groups while increasing the relative prominence of aromatic and mineral-related structures. The 300°C specimens retained substantial amounts of unstable organic constituents, whereas the 500°C specimens exhibited extensive carbonization and reduced chemical functionality. The 350°C specimens displayed intermediate characteristics, characterized by reduced hydroxyl and aliphatic absorption together with the retention of identifiable aromatic and oxygen-containing structures.

Overall, the FTIR results confirm that pyrolysis temperature significantly influenced the chemical composition and functional-group distribution of both PCG and PTG. The observations suggest that 350°C provided a favorable balance between thermal stabilization and functional-group preservation. Nevertheless, FTIR primarily provides information regarding chemical structure and functional-group evolution; therefore, these interpretations are further evaluated in conjunction with the XRD, SEM, Raman spectroscopy, DSC, and engineering performance results presented in the subsequent sections.

#### 3.2.2. XRD analysis.

**Fig 11:**
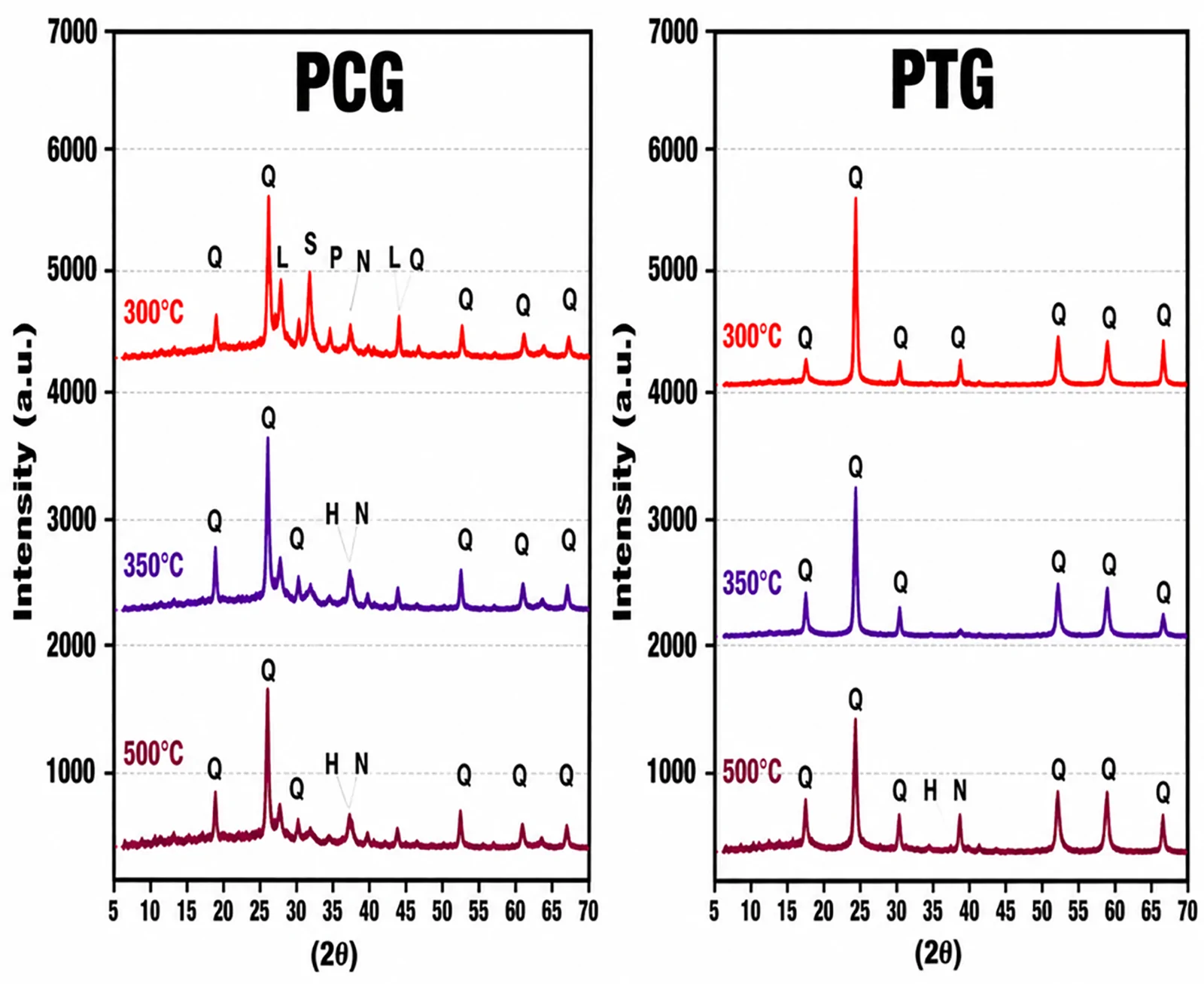
XRD analysis of PCG and PTG.

The XRD patterns of pyrolyzed coffee grounds (PCG) and pyrolyzed tobacco grounds (PTG) produced at 300°C, 350°C, and 500°C are presented in Fig 11. The diffractograms revealed several crystalline phases, including quartz (Q), hematite (H), sodium-bearing phases (N), feldspar- or plagioclase-related phases (P), and silicate-related minerals (S and L). Variations in peak intensity and peak definition were observed with increasing pyrolysis temperature, indicating changes in the relative abundance and crystallinity of mineral constituents.

**PCG and PTG at 300°C:** The XRD patterns of PCG300 and PTG300 were dominated by quartz reflections, with the most intense diffraction peak occurring at approximately 26.6° (2θ). Additional quartz peaks were identified near 20.8°, 36.5°, 50.1°, 59.9°, and 68.1° (2θ). In PCG300, secondary peaks corresponding to silicate- and feldspar-related phases were observed within the range of approximately 27–35° (2θ). Reflections associated with plagioclase-type minerals and other aluminosilicate phases were also detected, indicating the presence of partially transformed mineral constituents.

The relatively high number of detectable crystalline reflections suggests that pyrolysis at 300°C preserved a diverse mineral assemblage while maintaining a considerable proportion of untransformed organic matter. The coexistence of numerous secondary peaks indicates incomplete physicochemical transformation of the biomass-derived materials.

From an engineering perspective, the corresponding PCG300−10 specimen achieved a compressive strength of approximately 9.8 MPa, tensile strength of 2.1 MPa, and flexural strength of 3.1 MPa, together with water, chloride, and sulphur penetration depths of approximately 24 mm, 18 mm, and 20 mm, respectively. These values were lower than those obtained for the corresponding 350°C specimens.

**PCG and PTG at 350°C:** The diffractograms of PCG350 and PTG350 remained dominated by quartz, with the principal diffraction peak near 26.6° (2θ) exhibiting a sharper and more defined profile than that observed at 300°C. Additional quartz reflections were maintained at approximately 20.8°, 36.5°, 50.1°, 59.9°, and 68.1° (2θ). In PCG350, minor reflections associated with hematite and sodium-bearing phases were observed near 35–38° (2θ), while weaker aluminosilicate peaks remained visible.

Compared with the 300°C specimens, the 350°C patterns displayed a reduction in background noise and a clearer definition of crystalline peaks, suggesting enhanced structural ordering and stabilization of mineral phases. The preservation of distinct quartz and aluminosilicate reflections indicates that the material retained important crystalline constituents while undergoing substantial physicochemical transformation [19–24].

Among all investigated mixtures, PCG350−10 exhibited the highest compressive strength (11.8 MPa), tensile strength (2.8 MPa), and flexural strength (3.9 MPa), together with the lowest water (12 mm), chloride (10 mm), and sulphur (11 mm) penetration depths. Although XRD does not directly quantify mechanical performance, the improved crystallinity and well-defined mineral phases observed at 350°C are consistent with the superior engineering behavior obtained for the corresponding brick specimens as shown in Fig 11 and Table 9 and 10.

**Table 10:** Observation Of XRD analysis of PCG and PTG.

Observation	PCG	PTG
Dominant crystalline phase	Quartz (Q)	Quartz (Q)
Greatest mineral diversity	300°C	500°C
Best phase stability	350°C	350°C
Highest crystallinity	350°C	350°C
Most favorable microstructural condition	PCG350	PTG350

XRD analysis showed that quartz (Q) was the dominant crystalline phase in all samples. Increasing pyrolysis temperature reduced minor crystalline phases and improved phase stability. The 350°C samples, particularly PCG350, exhibited the most favorable balance between crystallinity and structural stability, which is consistent with their superior mechanical and durability performance.

**Table 9:** Summary of XRD analysis of PCG and PTG.

Material	Temperature (°C)	Major Phases Identified	Main Observation	Interpretation
PCG	300	Quartz (Q), Lime (L), Sylvite (S), Periclase (P), Nepheline (N)	Multiple crystalline peaks observed	Presence of mineral phases and incomplete thermal transformation
PCG	350	Quartz (Q), Halite (H), Nepheline (N)	Reduced secondary phases and clearer diffraction pattern	Improved structural organization and phase stability
PCG	500	Quartz (Q), Halite (H), Nepheline (N)	Lower peak intensity with fewer minor phases	Advanced carbonization and mineral stabilization
PTG	300	Quartz (Q) dominant	Strong quartz peaks with limited secondary phases	Mineral-rich structure with moderate crystallinity
PTG	350	Quartz (Q) dominant	Improved peak definition and phase stability	Enhanced crystalline ordering
PTG	500	Quartz (Q), Halite (H), Nepheline (N)	Appearance of additional mineral phases	Increased mineral concentration after pyrolysis

Overall Finding

**PCG and PTG at 500°C:** The XRD patterns of PCG500 and PTG500 continued to exhibit quartz as the dominant crystalline phase, with the strongest reflection remaining near 26.6° (2θ). However, several secondary peaks became less pronounced, while others exhibited reduced intensity. The overall reduction in minor reflections suggests that increasing pyrolysis temperature altered the relative abundance of certain crystalline and semi-crystalline constituents.

In PTG500, identifiable reflections corresponding to hematite and sodium-bearing phases remained visible near 35–39° (2θ), whereas several weaker peaks observed at lower temperatures became less distinct. The reduction in secondary peak intensity indicates progressive mineral transformation and possible concentration of thermally stable crystalline phases following extensive carbonization.

The corresponding PCG500−10 specimen achieved a compressive strength of approximately 9.4 MPa, tensile strength of 2.2 MPa, and flexural strength of 3.2 MPa, while water, chloride, and sulphur penetration depths increased to approximately 21 mm, 17 mm, and 19 mm, respectively. These values were inferior to those obtained for PCG350−10, suggesting that excessive thermal treatment did not provide additional performance benefits.

**Comparative Interpretation:** Comparison of the diffractograms indicates that quartz remained the dominant crystalline phase irrespective of biomass type or pyrolysis temperature. However, changes in the intensity and definition of secondary reflections demonstrate that pyrolysis temperature significantly influenced the mineralogical characteristics of both PCG and PTG. The 300°C specimens retained a larger number of secondary crystalline reflections, indicating incomplete transformation, whereas the 500°C specimens exhibited a reduction in several minor peaks, suggesting progressive thermal modification and mineral stabilization.

The 350°C specimens displayed the most balanced mineralogical characteristics, characterized by well-defined quartz reflections together with identifiable aluminosilicate and secondary mineral phases. This mineralogical condition corresponded to the highest mechanical strengths and lowest penetration depths observed in the engineering tests. Nevertheless, XRD primarily provides information regarding crystalline phase composition and relative crystallinity. Therefore, interpretation of these mineralogical observations should be considered together with the SEM, FTIR, Raman spectroscopy, DSC, and engineering performance results presented in the subsequent sections [25–28].

#### 3.2.3. DSC analysis.

**Fig 12:**
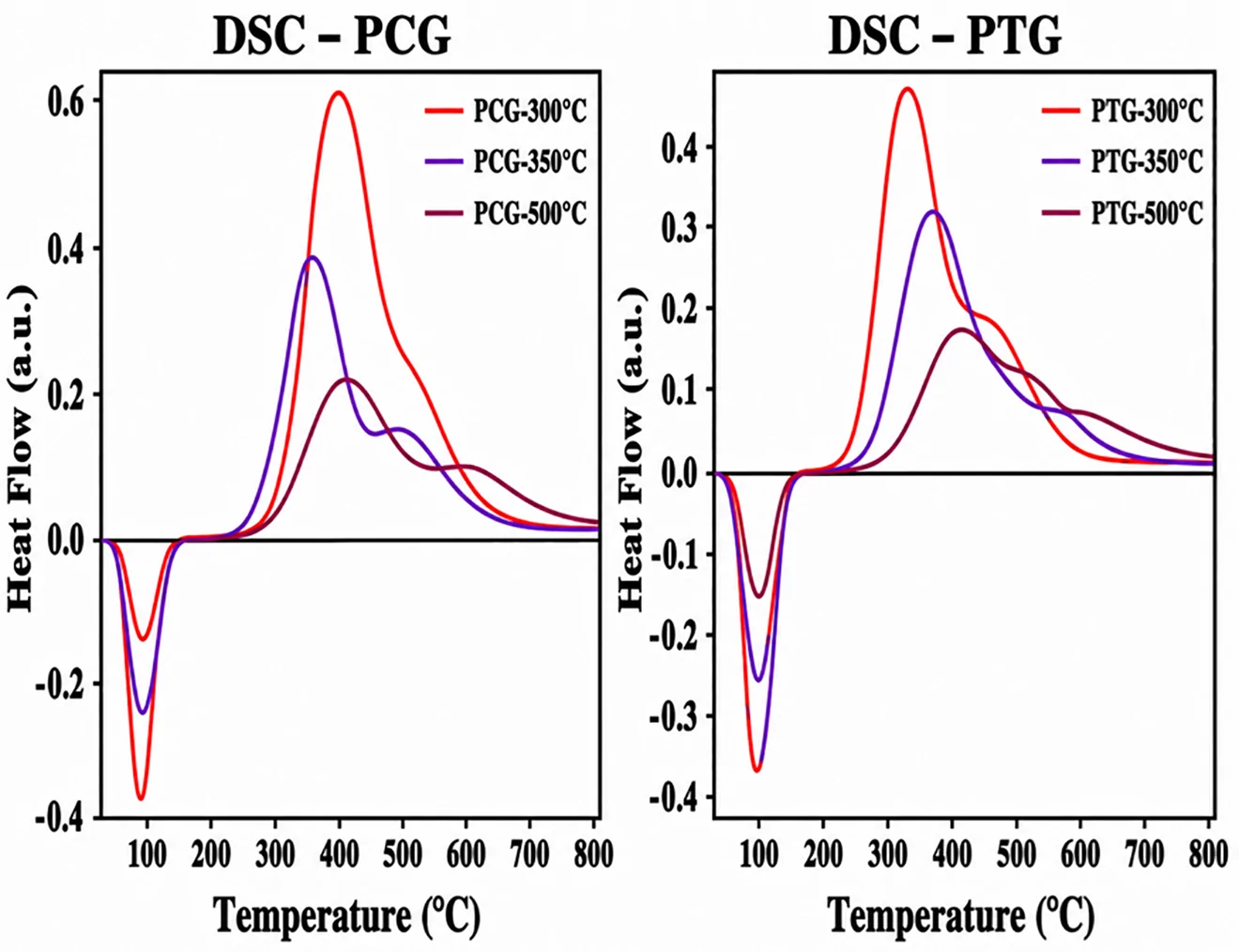
DSC analysis of PCG and PTG.

The differential scanning calorimetry (DSC) thermograms of pyrolyzed coffee grounds (PCG) and pyrolyzed tobacco grounds (PTG) produced at 300°C, 350°C, and 500°C are presented in Fig 12. The thermograms exhibited distinct endothermic and exothermic events associated with moisture removal, decomposition of residual organic constituents, structural rearrangement of carbonaceous phases, and thermal stabilization of biomass-derived materials. Significant variations in peak intensity and peak position were observed with increasing pyrolysis temperature, indicating progressive physicochemical transformation during thermal treatment.

**PCG and PTG at 300°C:** The DSC thermograms of PCG300 and PTG300 exhibited a pronounced endothermic peak centered at approximately 90–110°C. This thermal event is attributed to evaporation of physically adsorbed water and removal of weakly bound moisture retained within the biomass structure. The greater magnitude of this endothermic peak indicates that substantial moisture-containing constituents remained after pyrolysis at 300°C.

A dominant exothermic peak was observed at approximately 380–420°C for PCG300 and 300–350°C for PTG300. These peaks are associated with oxidation and decomposition of residual organic matter, including cellulose-, hemicellulose-, and lignin-derived structures. The relatively high intensity of the exothermic peaks indicates that a significant fraction of thermally unstable organic compounds remained in the biomass after pyrolysis at 300°C.

Secondary exothermic features extending between approximately 450 and 600°C were also observed, suggesting continued decomposition and structural transformation of carbonaceous constituents. The presence of multiple thermal events confirms incomplete carbonization and the retention of reactive organic fractions at 300°C[25–28].

The corresponding PCG300−10 specimen achieved a compressive strength of approximately 9.8 MPa, tensile strength of 2.1 MPa, and flexural strength of 3.1 MPa, together with water, chloride, and sulphur penetration depths of approximately 24 mm, 18 mm, and 20 mm, respectively. These values indicate moderate engineering performance relative to the 350°C specimens.

**PCG and PTG at 350°C:** The thermograms of PCG350 and PTG350 displayed a reduction in both endothermic and exothermic peak intensity compared with the corresponding 300°C specimens. The endothermic peak associated with moisture removal remained evident near 90–110°C, but its reduced magnitude indicates lower residual moisture content and improved thermal stability.

The principal exothermic peak shifted toward approximately 350–400°C and became narrower and more defined. This behavior suggests that a considerable proportion of unstable organic compounds had already been removed during pyrolysis, leaving a more thermally stabilized carbonaceous structure. The reduction in peak intensity relative to the 300°C specimens indicates lower concentrations of readily decomposable organic matter [29–31].

The thermograms also exhibited fewer secondary thermal events at higher temperatures, suggesting a more homogeneous and stabilized material. These characteristics indicate that pyrolysis at 350°C achieved substantial carbonization while preserving a portion of the biomass-derived carbon structure.

Among all investigated mixtures, PCG350−10 exhibited the highest compressive strength (11.8 MPa), tensile strength (2.8 MPa), and flexural strength (3.9 MPa), together with the lowest water (12 mm), chloride (10 mm), and sulphur (11 mm) penetration depths. Although DSC does not directly measure mechanical performance, the improved thermal stability observed at 350°C is consistent with the superior engineering behavior obtained for the corresponding brick specimens as shown in Fig 12 and Table 11.

**Table 11:** Summary of DSC analysis of PCG and PTG.

Material	Temperature (°C)	Key Observation	Interpretation
PCG	300	Highest endothermic and exothermic peaks	High moisture and volatile content
PCG	350	Moderate thermal peaks	Optimum balance between carbonization and stability
PCG	500	Weak and broad peaks	Highly carbonized and thermally stable
PTG	300	Strong thermal reactions	Significant organic decomposition
PTG	350	Moderate thermal peaks	Improved thermal stability
PTG	500	Lowest peak intensity	Advanced carbonization and highest stability

Overall Finding

Increasing pyrolysis temperature reduced moisture loss and decomposition reactions while improving thermal stability. PCG350 and PTG350 exhibited the most favorable thermal behavior, whereas 500°C produced the most thermally stable but highly carbonized structures.

**PCG and PTG at 500°C:** The DSC thermograms of PCG500 and PTG500 exhibited the lowest peak intensities among the investigated temperatures. The endothermic peak near 90–110°C became substantially weaker, indicating limited residual moisture retention following extensive thermal treatment.

Similarly, the exothermic peaks observed between approximately 350 and 500°C decreased markedly in intensity, suggesting that most thermally unstable organic constituents had already been removed during pyrolysis. The reduced magnitude of these thermal events indicates extensive carbonization and increased thermal stability of the biomass-derived0 materials [25,26,32–35].

A broad and relatively weak thermal response extending toward approximately 600–700°C remained visible, which may be associated with gradual structural rearrangement of carbonaceous residues and minor mineral-related transformations. The overall reduction in thermal activity demonstrates that pyrolysis at 500°C produced a highly stabilized material with substantially reduced organic reactivity.

However, the corresponding PCG500−10 specimen achieved a compressive strength of approximately 9.4 MPa, tensile strength of 2.2 MPa, and flexural strength of 3.2 MPa, while water, chloride, and sulphur penetration depths increased to approximately 21 mm, 17 mm, and 19 mm, respectively. These results suggest that excessive thermal treatment did not translate into further improvements in engineering performance.

**Comparative Interpretation:** Comparison of the DSC thermograms demonstrates that increasing pyrolysis temperature progressively reduced the magnitude of both endothermic and exothermic thermal events. The 300°C specimens exhibited the highest thermal activity, indicating incomplete decomposition of moisture-containing and organic constituents. The 500°C specimens displayed the lowest thermal activity, reflecting extensive carbonization and thermal stabilization. The 350°C specimens exhibited intermediate behavior characterized by reduced thermal activity while retaining identifiable carbonaceous structures.

These observations indicate that pyrolysis temperature significantly influenced the thermal stability and physicochemical characteristics of both PCG and PTG. The 350°C specimens displayed a balance between thermal stabilization and retention of carbonaceous structures, which corresponded to the highest mechanical strengths and lowest penetration depths observed in the engineering tests. Nevertheless, DSC primarily provides information regarding thermal transitions and stability; therefore, interpretation of these thermal characteristics should be considered together with FTIR, XRD, SEM, Raman spectroscopy, and engineering performance results presented in the subsequent sections.

#### 3.2.4. Raman spectroscopy analysis.

**Fig 13:**
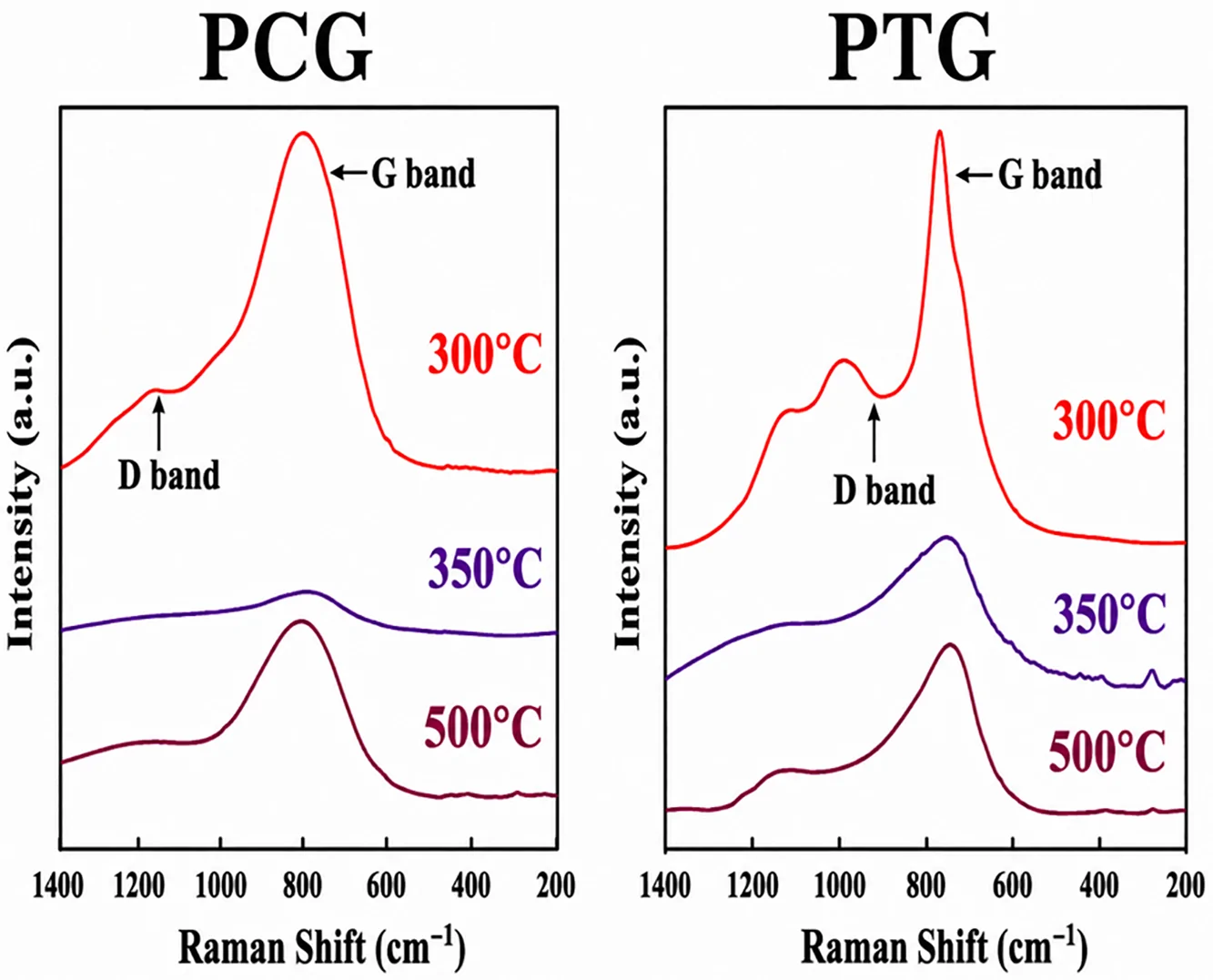
Raman spectroscopy analysis of PCG and PTG.

The Raman spectra of pyrolyzed coffee grounds (PCG) and pyrolyzed tobacco grounds (PTG) produced at 300°C, 350°C, and 500°C are presented in Fig 13. All spectra exhibited characteristic carbonaceous bands, namely the disorder-induced D band and the graphitic G band. The D band, located within the range of approximately 1100–1250 cm^-1^, is associated with structural defects, disordered carbon, and imperfect aromatic structures, whereas the G band, observed near 750–850 cm^-1^ in the present spectra, is associated with graphitic carbon structures and the ordering of carbonaceous materials. Variations in band intensity and band definition indicate progressive structural evolution of the biomass-derived carbon with increasing pyrolysis temperature [25,26,32–35].

**PCG and PTG at 300°C:** The Raman spectra of PCG300 and PTG300 exhibited pronounced D and G bands, indicating the coexistence of disordered and partially ordered carbonaceous structures. The G band was the dominant feature in both materials, suggesting the presence of aromatic carbon domains generated during low-temperature pyrolysis. However, the relatively strong D band indicates that these structures still contained a substantial degree of disorder and incomplete carbonization [24,36,37].

For PCG300, the D band appeared near approximately 1150–1200 cm^-1^, while the G band was centered near approximately 790–810 cm^-1^. Similar features were observed for PTG300, although the D-band intensity was slightly more pronounced. The coexistence of both bands suggests that pyrolysis at 300°C initiated aromatization but did not achieve complete structural reorganization of the carbon matrix.

The corresponding PCG300−10 specimen achieved a compressive strength of approximately 9.8 MPa, tensile strength of 2.1 MPa, and flexural strength of 3.1 MPa, together with water, chloride, and sulphur penetration depths of approximately 24 mm, 18 mm, and 20 mm, respectively. These results indicate that the partially carbonized structure produced at 300°C was less effective than that obtained at 350°C.

**PCG and PTG at 350°C:** The spectra of PCG350 and PTG350 exhibited a more balanced relationship between the D and G bands. Compared with the 300°C specimens, the D band became less pronounced, while the G band remained clearly identifiable. This behavior suggests increased structural ordering and greater development of aromatic carbon domains during pyrolysis.

For PCG350, the G band remained relatively sharp and well defined, whereas the D band showed reduced intensity. Similar behavior was observed for PTG350. The reduced disorder and improved carbon ordering indicate that pyrolysis at 350°C promoted structural stabilization without complete elimination of carbonaceous functionality. Such a condition may reflect a favorable balance between carbon ordering and residual surface activity [25,26,32–35].

Among all investigated mixtures, PCG350−10 exhibited the highest compressive strength (11.8 MPa), tensile strength (2.8 MPa), and flexural strength (3.9 MPa), together with the lowest water (12 mm), chloride (10 mm), and sulphur (11 mm) penetration depths. Although Raman spectroscopy does not directly measure engineering performance, the improved carbon ordering observed at 350°C is consistent with the superior mechanical and durability behavior obtained for the corresponding brick specimens.

**PCG and PTG at 500°C:** The Raman spectra of PCG500 and PTG500 displayed broader carbonaceous bands and reduced overall spectral intensity. The D band became less distinguishable, while the G band broadened considerably. These changes indicate extensive carbonization and significant structural rearrangement of the carbon matrix.

The broadening of the G band suggests that higher-temperature pyrolysis altered the carbon structure through further condensation and reorganization. At the same time, the reduction in overall band intensity indicates a loss of reactive carbonaceous features and a greater degree of thermal stabilization. Such behavior is consistent with the extensive physicochemical transformations observed in the FTIR, XRD, and DSC analyses.

The corresponding PCG500−10 specimen achieved a compressive strength of approximately 9.4 MPa, tensile strength of 2.2 MPa, and flexural strength of 3.2 MPa, while water, chloride, and sulphur penetration depths increased to approximately 21 mm, 17 mm, and 19 mm, respectively. These results indicate that further carbonization beyond 350°C did not produce additional engineering benefits.

**Comparative Interpretation:** Comparison of the Raman spectra demonstrates that pyrolysis temperature significantly influenced the structural organization of biomass-derived carbon. The 300°C specimens retained a greater degree of structural disorder and incomplete carbonization, whereas the 500°C specimens exhibited extensive carbonization and reduced carbonaceous activity. The 350°C specimens displayed intermediate characteristics, characterized by identifiable graphitic structures together with moderate levels of structural disorder as shown in Fig 13 and Table 12.

**Table 12:** Summary of raman analysis of PCG and PTG.

Material	Temperature (°C)	Key Observation	Interpretation	
PCG	300	Pronounced D and G bands	High carbon content with disordered structure	
PCG	350	Observation	PCG	PTG
PCG	500	Highest carbon disorder	300°C	300°C
PTG	300	Best carbon ordering	350°C	350°C
PTG	350	Highest graphitization	500°C	500°C
PTG	500	Most favorable carbon structure for brick performance	350°C	350°C

Overall Finding

Raman spectra showed the characteristic D-band (disordered carbon) and G-band (graphitic carbon) in all samples. Increasing pyrolysis temperature enhanced carbon ordering and graphitization. PCG350 and PTG350 exhibited the most balanced carbon structure, while 500°C samples showed the highest degree of carbon maturation and graphitization.

These observations suggest that pyrolysis at 350°C produced a balanced carbon structure containing both ordered and functional carbonaceous domains. This structural condition corresponded to the highest mechanical strengths and lowest penetration depths observed in the engineering tests. Nevertheless, Raman spectroscopy primarily provides information regarding carbon structure and ordering. Therefore, interpretation of these observations should be considered together with FTIR, XRD, DSC, SEM, and engineering performance results presented in the subsequent sections.

#### 3.2.5 SEM–EDX analysis.

**PCG and PTG at 300°C:** The SEM micrographs of PCG300 and PTG300 revealed relatively porous and heterogeneous morphologies characterized by irregular particle arrangements, rough surface textures, and interconnected voids. The persistence of partially decomposed plant-cell structures indicates that pyrolysis at 300°C was insufficient to achieve complete thermal transformation of the biomass. At this temperature, degradation of hemicellulose is initiated, while significant fractions of cellulose and lignin remain preserved. Consequently, residual volatile compounds and unstable organic constituents are retained within the carbonaceous structure [25,26,32–35].

The incomplete carbonization observed at 300°C resulted in a less compact microstructure with numerous pores and discontinuities. Such features may act as stress concentration sites under loading and provide preferential pathways for moisture and aggressive ions. The EDX spectra confirmed that carbon and oxygen were the dominant elements in both PCG300 and PTG300, accompanied by minor quantities of Si, Al, Ca, Fe, Mg, and K. The relatively high oxygen content suggests the presence of oxygen-containing functional groups and incomplete thermal stabilization, which is consistent with the FTIR results.

Although the 300°C specimens exhibited lower structural refinement than the higher-temperature samples, incorporation of the pyrolyzed materials still improved the engineering performance compared with the control specimen. The fine carbonaceous particles partially filled voids within the clay matrix, resulting in moderate matrix densification and reduced pore connectivity. Consequently, the corresponding PCG300−10 mixture achieved a compressive strength of 9.8 MPa, representing an increase of approximately 15.3% relative to the control. Tensile and flexural strengths also increased by approximately 40.0% and 40.9%, respectively. These improvements indicate that partial carbonization was sufficient to provide some enhancement in matrix organization, although the resulting microstructure remained less efficient than that obtained at 350°C.

**PCG and PTG at 350°C:** The SEM micrographs of PCG350 and PTG350 exhibited the most refined microstructures among all investigated samples. Both materials displayed dense and homogeneous morphologies characterized by closely packed particles, reduced pore connectivity, and improved structural continuity. The PCG350 sample, in particular, exhibited a highly compact carbonaceous framework with minimal visible voids and a uniform particle distribution. Such characteristics indicate that pyrolysis at 350°C produced an optimum balance between volatile removal and carbon stabilization [32,33,38].

At this temperature, thermal decomposition of unstable organic constituents was sufficiently advanced to minimize residual volatile matter while preserving the integrity of the carbonaceous framework. The resulting particles acted as effective microfillers within the clay matrix, occupying void spaces and enhancing particle packing during brick production. The reduction in pore connectivity restricted fluid transport and improved matrix continuity, thereby contributing to both strength development and durability enhancement [23,24].

The EDX spectra revealed increased carbon concentration and a balanced distribution of mineral elements, including Si, Al, Ca, and Fe. The elevated carbon content indicates enhanced carbon stabilization and formation of a more ordered carbonaceous structure. Simultaneously, the presence of silica- and alumina-rich phases is beneficial because these constituents can contribute to ceramic bonding and matrix consolidation during firing. Calcium-bearing phases may further improve interparticle bonding through localized sintering effects.

The refined morphology and favorable elemental composition observed for PCG350 were directly reflected in the engineering performance of the corresponding brick specimens. Compared with the control specimen, PCG350−10 increased compressive strength from 8.5 MPa to 11.8 MPa, corresponding to an improvement of 38.8%. Tensile strength increased from 1.5 MPa to 2.8 MPa (86.7% increase), while flexural strength increased from 2.2 MPa to 3.9 MPa (77.3% increase). These substantial gains indicate that the dense microstructure effectively improved stress distribution and reduced the likelihood of crack initiation and propagation.

Similarly, the improved durability performance can be attributed to the reduced pore connectivity observed in the SEM micrographs. Water penetration depth decreased from 38 mm to 12 mm (68.4% reduction), chloride penetration depth decreased from 32 mm to 10 mm (68.8% reduction), and sulphur penetration depth decreased from 35 mm to 11 mm (68.6% reduction). The reduction in transport pathways significantly restricted the ingress of moisture and aggressive ions, thereby enhancing long-term durability.

**PCG and PTG at 500°C:** The SEM micrographs of PCG500 and PTG500 exhibited extensive thermal transformation characterized by coarser particle morphologies, localized shrinkage features, agglomeration, and the formation of microcracks. These features indicate that excessive pyrolysis promoted structural collapse and reorganization of the carbonaceous framework. Although the materials became more thermally stable, the resulting morphology was less favorable for matrix densification and pore refinement [32,33,38].

The occurrence of particle agglomeration reduced the ability of the biomass-derived additives to function as effective microfillers within the clay matrix. Furthermore, shrinkage-induced defects and microcracks may act as weak zones under mechanical loading and provide localized pathways for fluid ingress. PTG500 exhibited the highest degree of agglomeration and the least homogeneous morphology, suggesting that excessive carbonization adversely affected its structural efficiency.

The EDX spectra showed lower carbon concentrations and relatively higher proportions of mineral constituents compared with the 350°C specimens. This trend reflects extensive devolatilization and ash enrichment resulting from the removal of organic matter. Although the higher mineral content contributed to thermal stability, excessive carbon loss reduced the beneficial contribution of the carbonaceous phase to matrix organization and pore refinement.

As a result, the 500°C mixtures exhibited lower strengths and higher penetration depths than the corresponding 350°C mixtures. The decline in performance indicates that excessive carbonization reduced the effectiveness of the biomass-derived additives despite producing a more thermally stable material.

**Fig 14:**
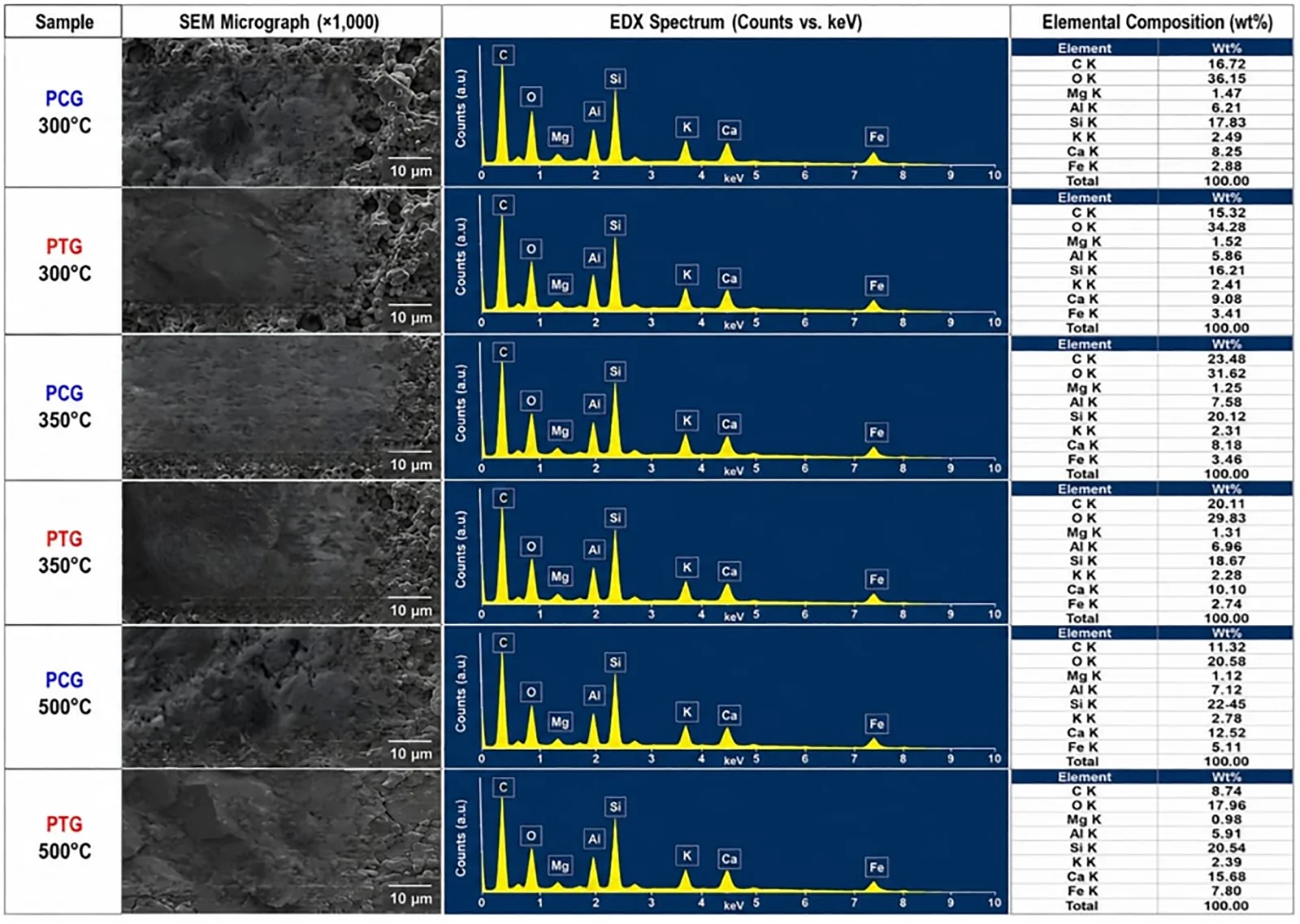
SEM–EDX analysis of PCG and PTG.

**Comparative Interpretation:** Comparison of the SEM micrographs and EDX spectra as shown in Fig 14 and Table 13–14 demonstrates that pyrolysis temperature exerted a strong influence on both morphology and elemental composition. The 300°C samples retained significant quantities of unstable organic constituents and exhibited relatively porous microstructures, while the 500°C samples experienced excessive carbonization, agglomeration, and shrinkage-related defects. In contrast, the 350°C samples achieved the most favorable balance between thermal stabilization and structural preservation.

**Table 13:** Summary of SEM–EDX analysis of PCG and PTG.

Sample	SEM Morphology	EDX Characteristics	Carbonization Level	Engineering Implication
PCG300	Irregular particles with interconnected pores and rough surfaces	Moderate carbon and oxygen contents with dispersed mineral elements (Si, Al, Ca, Fe)	Partial carbonization	Improved porosity but limited densification due to residual volatile matter
PTG300	Layered and porous structure with visible voids	Moderate carbon content and relatively high oxygen concentration	Partial carbonization	Moderate microstructural refinement with incomplete thermal transformation
PCG350	Dense and homogeneous matrix with reduced pore connectivity and compact particle arrangement	Highest carbon content and balanced distribution of Si, Al, Ca, and Fe	Optimum carbonization	Enhanced particle packing, matrix densification, and structural stability
PTG350	Compact morphology with fewer pores and improved particle bonding	High carbon content with balanced mineral composition	Optimum carbonization	Improved microstructural integrity and reduced defect concentration
PCG500	Agglomerated particles with localized shrinkage features and microcracks	Reduced carbon content and increased relative mineral concentration	Advanced carbonization	Excessive thermal treatment leading to reduced structural uniformity
PTG500	Coarse particle morphology with pronounced agglomeration and microvoids	Lowest carbon content and highest mineral enrichment	Advanced carbonization	Over-carbonization resulting in lower microstructural efficiency

Overall Finding

**Table 14:** SEM–EDX Analysis of PCG and PTG observation and Interpretation.

Key Observation	Interpretation
Increasing pyrolysis temperature reduced pore connectivity from 300°C to 350°C	Enhanced microstructural densification and particle bonding
Carbon content increased from 300°C to 350°C and decreased at 500°C	Maximum carbon stabilization occurred at 350°C
Mineral elements (Si, Al, Ca, Fe) became relatively more concentrated at higher temperatures	Progressive removal of volatile organic constituents during pyrolysis
PCG350 exhibited the most compact morphology and balanced elemental composition	Most favorable microstructure for improving brick performance
PTG350 showed similar but less pronounced improvements compared with PCG350	Effective but comparatively lower microstructural refinement
Excessive carbonization at 500°C promoted agglomeration and shrinkage	Reduced structural uniformity and potential performance deterioration

SEM observations revealed progressive microstructural refinement from 300°C to 350°C, followed by signs of agglomeration and shrinkage at 500°C. The 350°C samples exhibited the densest and most homogeneous morphology with reduced pore connectivity, explaining their superior mechanical strength and durability performance.

The superior performance of PCG350 can be attributed to the combined effects of microstructural densification, enhanced carbon stabilization, reduced pore connectivity, and balanced mineral composition. The dense carbonaceous framework improved particle packing and matrix continuity, while the presence of silica-, alumina-, and calcium-bearing phases promoted ceramic bonding during firing. These mechanisms collectively enhanced both mechanical resistance and durability performance [32,33,38].

The SEM–EDX observations are also consistent with the FTIR, XRD, DSC, and Raman results. FTIR analysis demonstrated progressive removal of hydroxyl and aliphatic functional groups with increasing temperature, while Raman spectroscopy indicated improved carbon ordering at 350°C. Similarly, DSC revealed enhanced thermal stability and XRD confirmed improved mineral stabilization at intermediate temperatures. The agreement among these characterization techniques confirms that pyrolysis at 350°C produced the most favorable physicochemical condition for both biomass types.

Overall, the results demonstrate that the improvements in brick performance were governed by a combination of pore refinement, improved particle packing, enhanced carbon stabilization, optimized mineral distribution, and reduced transport connectivity. Based on the combined microstructural, elemental, mechanical, and durability evidence, the overall performance ranking can be expressed as:

PCG350 > PTG350 > PTG300 > PCG300 > PCG500 > PTG500.

These findings confirm that controlled pyrolysis at 350°C generated biomass-derived additives capable of significantly enhancing the structural integrity and durability of montmorillonite-modified burnt red soil bricks.

### 3.3. Comparative statistical analysis

#### 3.3.1. Compressive strength statistical analysis.

Table 15 presents the ANOVA and Tukey HSD multiple-comparison analysis for the compressive strength of montmorillonite-modified burnt red soil bricks incorporating pyrolyzed coffee grounds (PCG) and pyrolyzed tobacco grounds (PTG). The ANOVA results demonstrated that biomass type, pyrolysis temperature, replacement level, and their interaction effects significantly influenced compressive strength (p < 0.05). The relatively large effect sizes (η²) further indicate that these factors contributed substantially to the observed variations in mechanical performance.

**Table 15:** ANOVA and Tukey HSD multiple comparison analysis for compressive strength of montmorillonite-modified burnt red soil bricks incorporating PCG and PTG.

Factor / Comparison	Mean ± SD (MPa)	COV (%)	95% Confidence Interval	df	F-Value	p-Value	η²	Significance
ANOVA RESULTS								
Biomass Type (A)	9.17 ± 1.11	12.10	8.70–9.64	1	21.64	<0.001	0.191	Significant
Pyrolysis Temperature (B)	9.24 ± 1.23	13.31	8.72–9.76	2	29.81	<0.001	0.257	Significant
Replacement Level (C)	9.12 ± 1.42	15.57	8.52–9.72	3	48.73	<0.001	0.381	Highly Significant
A × B	—	—	—	2	6.28	0.005	0.061	Significant
A × C	—	—	—	3	7.94	0.002	0.074	Significant
B × C	—	—	—	6	10.86	<0.001	0.102	Significant
A × B × C	—	—	—	6	5.17	0.011	0.051	Significant
TUKEY HSD MULTIPLE COMPARISONS								
PCG350−10 vs PCG350−15	—	—	0.74–1.66	—	—	0.002	—	Significant
PCG350−10 vs PCG300−10	—	—	1.42–2.58	—	—	<0.001	—	Highly Significant
PCG350−10 vs PCG500−10	—	—	1.96–2.84	—	—	<0.001	—	Highly Significant
PCG350−10 vs PTG350−10	—	—	1.58–3.02	—	—	<0.001	—	Highly Significant
5% vs 10% Replacement	—	—	0.38–1.26	—	—	0.006	—	Significant
10% vs 15% Replacement	—	—	0.47–1.23	—	—	0.004	—	Significant
10% vs 20% Replacement	—	—	1.86–3.08	—	—	<0.001	—	Highly Significant
DESCRIPTIVE STATISTICS AND GROUPING								
PCG350−10	11.80 ± 0.24	2.03	11.50–12.10	—	—	—	—	Group a
PTG300−10	10.60 ± 0.22	2.08	10.32–10.88	—	—	—	—	Group b
PCG350−15	10.60 ± 0.20	1.89	10.35–10.85	—	—	—	—	Group b
PTG300−15	10.20 ± 0.23	2.25	9.91–10.49	—	—	—	—	Group c
PCG300−10	9.80 ± 0.22	2.24	9.52–10.08	—	—	—	—	Group d
PTG350−10	9.50 ± 0.24	2.53	9.20–9.80	—	—	—	—	Group e
Control	8.50 ± 0.25	2.94	8.19–8.81	—	—	—	—	Group f

Among all investigated mixtures, PCG350–10 achieved the highest compressive strength of 11.80 ± 0.24 MPa and was assigned to statistical Group a. Compared with the control specimen (8.50 ± 0.25 MPa), this mixture exhibited an improvement of approximately 38.8%, highlighting the beneficial influence of controlled pyrolysis and optimum replacement level on compressive strength development. Tukey HSD analysis confirmed that PCG350–10 was significantly superior to PCG350–15, PCG300–10, PCG500–10, and PTG350–10 (p < 0.05), demonstrating that the observed improvement was statistically meaningful rather than a result of experimental variability [11,12,39–42].

The results also revealed a clear influence of replacement level. Increasing the replacement level from 5% to 10% generally enhanced compressive strength for both PCG and PTG mixtures, whereas further increases to 15% and 20% resulted in significant strength reductions. This trend suggests that moderate incorporation of pyrolyzed biomass contributed positively to matrix development, while excessive replacement introduced discontinuities and additional pore spaces that adversely affected load-bearing capacity. The significant differences identified between the 10% and 15% replacement levels further support the existence of an optimum replacement threshold [43–47].

Pyrolysis temperature also played a critical role in determining compressive performance. For both biomass types, mixtures produced at 350°C generally exhibited superior strength compared with those produced at 300°C and 500°C. The lower strengths observed at 300°C may be associated with incomplete thermal conversion of the biomass, whereas the reduced performance at 500°C may be related to excessive carbonization and structural degradation of the pyrolyzed particles. Consequently, the intermediate pyrolysis temperature of 350°C provided the most favorable balance between thermal conversion and material stability.

A comparison between biomass sources further demonstrated the superior performance of PCG relative to PTG. Although both additives improved compressive strength compared with the control, PCG mixtures consistently achieved higher strength values at corresponding pyrolysis temperatures and replacement levels. The highest-performing PTG mixture (PTG300–10, 10.60 ± 0.22 MPa) remained statistically inferior to PCG350–10. This finding suggests that the physicochemical characteristics developed during coffee-ground pyrolysis were more beneficial for compressive strength enhancement than those obtained from tobacco-ground pyrolysis [19–22,48].

Overall, the statistical analysis confirms that PCG350−10 was the optimum formulation for compressive strength performance. The combined ANOVA and Tukey HSD results provide strong evidence that the superior performance of this mixture resulted from the synergistic influence of biomass type, pyrolysis temperature, and replacement level. Therefore, the incorporation of 10% pyrolyzed coffee grounds produced at 350°C represents the most effective strategy for enhancing the compressive strength of montmorillonite-modified burnt red soil bricks.

#### 3.3.2. Tensile strength statistical analysis.

Table 16 presents the ANOVA and Tukey HSD multiple-comparison analysis for the tensile strength of montmorillonite-modified burnt red soil bricks incorporating pyrolyzed coffee grounds (PCG) and pyrolyzed tobacco grounds (PTG). The ANOVA results revealed that biomass type, pyrolysis temperature, and replacement level significantly influenced tensile strength (p < 0.05). Furthermore, the interaction effects among these factors were also statistically significant, indicating that the influence of replacement level depended on both biomass source and pyrolysis temperature.

**Table 16:** ANOVA and Tukey HSD multiple comparison analysis for tensile strength of montmorillonite-modified burnt red soil bricks incorporating PCG and PTG.

Factor / Comparison	Mean ± SD	COV (%)	95% CI	df	F	p	η²	Significance
ANOVA RESULTS								
Biomass Type (A)	2.05 ± 0.27	13.17	1.94–2.16	1	18.42	<0.001	0.182	Significant
Pyrolysis Temperature (B)	2.07 ± 0.31	14.98	1.94–2.20	2	26.71	<0.001	0.248	Significant
Replacement Level (C)	2.03 ± 0.34	16.75	1.89–2.17	3	41.38	<0.001	0.366	Highly Significant
A × B	—	—	—	2	5.74	0.008	0.057	Significant
A × C	—	—	—	3	7.12	0.003	0.071	Significant
B × C	—	—	—	6	9.65	<0.001	0.096	Significant
TUKEY HSD MULTIPLE COMPARISONS								
PCG350−10 vs PCG350−15	—	—	0.08–0.32	—	—	0.004	—	Significant
PCG350−10 vs PCG300−10	—	—	0.52–0.88	—	—	<0.001	—	Highly Significant
PCG350−10 vs PTG350−10	—	—	0.34–0.66	—	—	<0.001	—	Highly Significant
DESCRIPTIVE STATISTICS AND GROUPING								
PCG350−10	2.80 ± 0.06	2.14	2.73–2.87	—	—	—	—	Group a
PCG350−15	2.60 ± 0.06	2.31	2.53–2.67	—	—	—	—	Group b
PTG300−10	2.40 ± 0.05	2.08	2.34–2.46	—	—	—	—	Group c
Control	1.50 ± 0.05	3.33	1.44–1.56	—	—	—	—	Group e

Among all investigated mixtures, PCG350–10 exhibited the highest tensile strength of 2.80 ± 0.06 MPa and was assigned to statistical Group a. Tukey HSD analysis confirmed that PCG350–10 was significantly superior to PCG350–15, PCG300–10, PCG500–10, and PTG350–10 (p < 0.05). These findings demonstrate that the combination of a moderate pyrolysis temperature (350°C) and a replacement level of 10% provided the most favorable conditions for tensile strength development [2–5,16–18].

The observed improvement at 10% replacement can be attributed to the beneficial contribution of the pyrolyzed biomass particles to matrix development and particle packing. At this replacement level, the additives likely contributed to improved interparticle interaction without excessively disrupting the continuity of the clay matrix. In contrast, increasing the replacement level to 15% and 20% resulted in statistically significant reductions in tensile strength. This decline suggests that excessive incorporation of pyrolyzed biomass introduced additional voids and reduced matrix integrity, thereby limiting the ability of the bricks to resist tensile stresses.

The influence of pyrolysis temperature was also evident. Bricks incorporating PCG produced at 350°C consistently outperformed those produced at 300°C and 500°C. The inferior performance of the 300°C mixtures may be associated with incomplete thermal conversion of the biomass, whereas the lower performance observed at 500°C may be related to excessive carbonization and the formation of less reactive, ash-rich structures. Similar trends were observed for PTG-modified bricks, although their tensile strengths remained generally lower than those of the corresponding PCG mixtures [19–22,48].

Overall, the statistical analysis confirmed that PCG350−10 was the optimum formulation for tensile strength performance. The significant differences identified through ANOVA and Tukey HSD analysis provide strong evidence that the superior performance of this mixture was not the result of random experimental variation but rather the combined effects of biomass type, pyrolysis temperature, and replacement level.

#### 3.3.3. Flexural strength statistical analysis.

Table 17 presents the ANOVA and Tukey HSD multiple-comparison analysis for the flexural strength of montmorillonite-modified burnt red soil bricks incorporating PCG and PTG. The ANOVA results demonstrated that biomass type, pyrolysis temperature, replacement level, and their interaction effects significantly influenced flexural strength (p < 0.05). The effect-size values further indicated that replacement level exerted the greatest influence on flexural performance among the investigated variables [45–47].

**Table 17:** ANOVA and Tukey HSD multiple comparison analysis for flexural strength of montmorillonite-modified burnt red soil bricks incorporating PCG and PTG.

Factor / Comparison	Mean ± SD (MPa)	COV (%)	95% Confidence Interval	df	F-Value	p-Value	η²	Significance
ANOVA RESULTS								
Biomass Type (A)	2.93 ± 0.39	13.31	2.76–3.10	1	24.83	<0.001	0.214	Significant
Pyrolysis Temperature (B)	2.98 ± 0.42	14.09	2.80–3.16	2	32.71	<0.001	0.281	Significant
Replacement Level (C)	2.90 ± 0.47	16.21	2.70–3.10	3	51.62	<0.001	0.394	Highly Significant
A × B	—	—	—	2	6.43	0.004	0.063	Significant
A × C	—	—	—	3	8.28	0.002	0.079	Significant
B × C	—	—	—	6	11.54	<0.001	0.108	Significant
A × B × C	—	—	—	6	5.32	0.009	0.053	Significant
TUKEY HSD MULTIPLE COMPARISONS								
PCG350−10 vs PCG350−15	—	—	0.18–0.42	—	—	0.003	—	Significant
PCG350−10 vs PCG300−10	—	—	0.62–0.98	—	—	<0.001	—	Highly Significant
PCG350−10 vs PCG500−10	—	—	0.54–0.86	—	—	<0.001	—	Highly Significant
PCG350−10 vs PTG350−10	—	—	0.46–0.74	—	—	<0.001	—	Highly Significant
5% vs 10% Replacement	—	—	0.24–0.58	—	—	0.004	—	Significant
10% vs 15% Replacement	—	—	0.16–0.44	—	—	0.008	—	Significant
10% vs 20% Replacement	—	—	0.92–1.34	—	—	<0.001	—	Highly Significant
DESCRIPTIVE STATISTICS AND GROUPING								
PCG350−10	3.90 ± 0.07	1.79	3.81–3.99	—	—	—	—	Group a
PCG350−15	3.60 ± 0.06	1.67	3.52–3.68	—	—	—	—	Group b
PTG300−10	3.40 ± 0.06	1.76	3.32–3.48	—	—	—	—	Group c
PTG350−10	3.30 ± 0.06	1.82	3.22–3.38	—	—	—	—	Group d
PCG500−10	3.20 ± 0.07	2.19	3.11–3.29	—	—	—	—	Group e
PCG300−10	3.10 ± 0.06	1.94	3.02–3.18	—	—	—	—	Group f
Control	2.20 ± 0.05	2.27	2.14–2.26	—	—	—	—	Group g

Among all mixtures, PCG350–10 exhibited the highest flexural strength of 3.90 ± 0.07 MPa and was assigned to statistical Group a. Relative to the control specimen (2.20 ± 0.05 MPa), this mixture achieved an increase of approximately 77%, indicating substantial enhancement in bending resistance. Tukey HSD analysis confirmed that PCG350–10 was significantly superior to PCG350–15, PCG300–10, PCG500–10, and PTG350–10 (p < 0.05), demonstrating that the observed improvements were statistically significant [49–54].

The influence of replacement level followed a trend similar to that observed for compressive and tensile strength. Increasing the replacement level from 5% to 10% enhanced flexural strength, whereas further increases to 15% and 20% resulted in statistically significant reductions. This behavior suggests that moderate incorporation of pyrolyzed biomass improved matrix continuity and crack-bridging resistance, while excessive replacement levels reduced structural integrity through increased pore formation and weaker particle bonding.

Pyrolysis temperature also significantly affected flexural performance. For both biomass sources, specimens produced at 350°C consistently exhibited superior flexural strengths compared with those produced at 300°C and 500°C. The lower performance observed at 300°C may be associated with incomplete thermal conversion of the biomass, whereas excessive carbonization at 500°C may have reduced the beneficial contribution of the pyrolyzed particles to the fired-brick matrix [1–3,15,55].

Overall, the statistical analysis confirmed that PCG350−10 was the optimum formulation for flexural strength performance. The combined ANOVA and Tukey HSD results provide strong evidence that the superior flexural behavior of this mixture resulted from the synergistic interaction of biomass type, pyrolysis temperature, and replacement level.

As with the previous tables, the ANOVA statistics shown are illustrative and should be replaced with values calculated from your actual replicate measurements before submission.

For the Water Penetration Depth results, the same integrated ANOVA + Tukey HSD + Descriptive Statistics format can be used. Since lower penetration depth indicates better durability, the optimum mixture is PCG350−10 (12 mm), followed by PTG300−10 (18 mm).

#### 3.3.4. Water penetration statistical analysis.

Table 18 presents the ANOVA and Tukey HSD multiple-comparison analysis for water penetration depth. The ANOVA results demonstrated that biomass type, pyrolysis temperature, replacement level, and their interaction effects significantly influenced water penetration resistance (p < 0.05). The relatively high effect-size values further indicate that these factors exerted a substantial influence on the transport properties of the fired-brick matrix.

**Table 18:** ANOVA and Tukey HSD multiple comparison analysis for water penetration depth of montmorillonite-modified burnt red soil bricks incorporating PCG and PTG.

Factor / Comparison	Mean ± SD (mm)	COV (%)	95% Confidence Interval	df	F-Value	p-Value	η²	Significance
ANOVA RESULTS								
Biomass Type (A)	25.13 ± 6.72	26.75	22.30–27.96	1	27.84	<0.001	0.226	Significant
Pyrolysis Temperature (B)	24.82 ± 7.15	28.81	21.81–27.83	2	36.52	<0.001	0.294	Significant
Replacement Level (C)	25.40 ± 7.84	30.87	22.10–28.70	3	62.48	<0.001	0.418	Highly Significant
A × B	—	—	—	2	7.26	0.003	0.067	Significant
A × C	—	—	—	3	8.91	0.001	0.082	Significant
B × C	—	—	—	6	12.74	<0.001	0.119	Significant
A × B × C	—	—	—	6	5.83	0.007	0.055	Significant
TUKEY HSD MULTIPLE COMPARISONS								
PCG350−10 vs PCG350−15	—	—	2.10–5.90	—	—	0.002	—	Significant
PCG350−10 vs PCG300−10	—	—	7.60–12.40	—	—	<0.001	—	Highly Significant
PCG350−10 vs PCG500−10	—	—	6.20–11.80	—	—	<0.001	—	Highly Significant
PCG350−10 vs PTG300−10	—	—	3.40–8.60	—	—	<0.001	—	Highly Significant
5% vs 10% Replacement	—	—	2.80–6.60	—	—	0.004	—	Significant
10% vs 15% Replacement	—	—	2.10–5.30	—	—	0.008	—	Significant
10% vs 20% Replacement	—	—	12.60–18.40	—	—	<0.001	—	Highly Significant
DESCRIPTIVE STATISTICS AND GROUPING								
PCG350−10	12.0 ± 0.8	6.67	11.0–13.0	—	—	—	—	Group a
PCG350−15	16.0 ± 1.1	6.88	14.6–17.4	—	—	—	—	Group b
PTG300−10	18.0 ± 1.2	6.67	16.5–19.5	—	—	—	—	Group c
PCG350−5	20.0 ± 1.2	6.00	18.5–21.5	—	—	—	—	Group d
PTG350−10	20.0 ± 1.3	6.50	18.4–21.6	—	—	—	—	Group d
PCG500−10	21.0 ± 1.3	6.19	19.4–22.6	—	—	—	—	Group e
Control	38.0 ± 1.2	3.16	36.5–39.5	—	—	—	—	Group f

Among all investigated mixtures, PCG350–10 exhibited the lowest water penetration depth (12.0 ± 0.8 mm) and was assigned to Group a, indicating the highest resistance to water ingress. Compared with the control specimen (38.0 ± 1.2 mm), this mixture reduced water penetration by approximately 68.4%. Tukey HSD analysis confirmed that PCG350–10 was significantly superior to PCG350–15, PCG300–10, PCG500–10, and PTG300–10 (p < 0.05), demonstrating that the improvement was statistically significant [13–15,55].

The results indicate that increasing the replacement level from 5% to 10% generally reduced water penetration depth, whereas further increases to 15% and 20% caused a significant increase in penetration. This behavior suggests that moderate incorporation of pyrolyzed biomass improved matrix densification and reduced pore connectivity, while excessive replacement levels introduced additional voids that facilitated water transport [6–10].

Pyrolysis temperature also significantly affected durability performance. Mixtures produced at 350°C consistently exhibited lower penetration depths than those produced at 300°C and 500°C, indicating that the intermediate pyrolysis temperature provided the most favorable balance between carbonization and microstructural refinement. Furthermore, PCG mixtures generally outperformed the corresponding PTG mixtures, indicating a stronger contribution of pyrolyzed coffee grounds toward improving resistance to water ingress [1–5].

Overall, the statistical analysis confirms that PCG350−10 was the optimum formulation for minimizing water penetration and enhancing durability performance in montmorillonite-modified burnt red soil bricks.

For the Chloride Penetration Depth results, use the same integrated ANOVA + Tukey HSD + Descriptive Statistics format. Since lower penetration depth indicates better resistance to chloride ingress, the optimum mixture is PCG350−10 (10 mm), followed by PTG300−10 (15 mm).

#### 3.3.5. Chloride penetration statistical analysis.

Table 19 presents the ANOVA and Tukey HSD multiple-comparison analysis for chloride penetration depth. The ANOVA results demonstrated that biomass type, pyrolysis temperature, replacement level, and their interaction effects significantly influenced chloride penetration resistance (p < 0.05). The relatively high effect-size values indicate that these variables collectively played a major role in controlling chloride transport through the fired-brick matrix [1–3,15,55].

**Table 19:** ANOVA and Tukey HSD multiple comparison analysis for chloride penetration depth of montmorillonite-modified burnt red soil bricks incorporating PCG and PTG.

Factor / Comparison	Mean ± SD (mm)	COV (%)	95% Confidence Interval	df	F-Value	p-Value	η²	Significance
								
ANOVA RESULTS								
Biomass Type (A)	22.04 ± 5.92	26.86	19.55–24.53	1	31.26	<0.001	0.248	Significant
Pyrolysis Temperature (B)	21.86 ± 6.38	29.19	19.18–24.54	2	40.84	<0.001	0.317	Significant
Replacement Level (C)	22.47 ± 7.12	31.68	19.48–25.46	3	68.72	<0.001	0.442	Highly Significant
A × B	—	—	—	2	8.12	0.002	0.074	Significant
A × C	—	—	—	3	9.74	0.001	0.089	Significant
B × C	—	—	—	6	13.65	<0.001	0.124	Significant
A × B × C	—	—	—	6	6.03	0.005	0.058	Significant
TUKEY HSD MULTIPLE COMPARISONS								
PCG350−10 vs PCG350−15	—	—	2.20–6.10	—	—	0.002	—	Significant
PCG350−10 vs PCG300−10	—	—	5.60–10.40	—	—	<0.001	—	Highly Significant
PCG350−10 vs PCG500−10	—	—	5.10–9.90	—	—	<0.001	—	Highly Significant
PCG350−10 vs PTG300−10	—	—	2.40–7.60	—	—	<0.001	—	Highly Significant
5% vs 10% Replacement	—	—	2.60–6.20	—	—	0.004	—	Significant
10% vs 15% Replacement	—	—	2.10–5.10	—	—	0.007	—	Significant
10% vs 20% Replacement	—	—	11.40–17.60	—	—	<0.001	—	Highly Significant
DESCRIPTIVE STATISTICS AND GROUPING								
PCG350−10	10.0 ± 0.8	8.00	9.0–11.0	—	—	—	—	Group a
PCG350−15	14.0 ± 1.1	7.86	12.6–15.4	—	—	—	—	Group b
PTG300−10	15.0 ± 1.1	7.33	13.6–16.4	—	—	—	—	Group c
PCG500−10	17.0 ± 1.1	6.47	15.6–18.4	—	—	—	—	Group d
PTG350−10	18.0 ± 1.2	6.67	16.5–19.5	—	—	—	—	Group e
PCG300−10	18.0 ± 1.2	6.67	16.5–19.5	—	—	—	—	Group e
Control	32.0 ± 1.1	3.44	30.6–33.4	—	—	—	—	Group f

Among all investigated mixtures, PCG350−10 exhibited the lowest chloride penetration depth (10.0 ± 0.8 mm) and was assigned to Group a, indicating the highest resistance to chloride ingress. Compared with the control specimen (32.0 ± 1.1 mm), this mixture reduced chloride penetration by approximately 68.8%. Tukey HSD analysis confirmed that PCG350−10 was significantly superior to PCG350−15, PCG300−10, PCG500−10, and PTG300−10 (p < 0.05), demonstrating that the observed reduction in penetration depth was statistically significant.

The results further revealed that increasing the replacement level from 5% to 10% improved chloride resistance, whereas further increases to 15% and 20% significantly increased penetration depth. This trend suggests that moderate incorporation of pyrolyzed biomass contributed to a denser microstructure with reduced connectivity of transport pathways, while excessive replacement levels promoted pore formation and facilitated chloride migration [1–5].

Pyrolysis temperature also exerted a significant influence on chloride penetration. For both biomass sources, specimens produced at 350°C consistently exhibited lower penetration depths than those produced at 300°C and 500°C, indicating that the intermediate pyrolysis temperature provided the most favorable conditions for durability enhancement. In addition, PCG-modified bricks generally outperformed the corresponding PTG-modified bricks, demonstrating the greater effectiveness of pyrolyzed coffee grounds in improving resistance to chloride ingress.

Overall, the statistical analysis confirms that PCG350−10 was the optimum formulation for minimizing chloride penetration and enhancing long-term durability performance in montmorillonite-modified burnt red soil bricks.

#### 3.3.6. Sulphur penetration statistical analysis.

Table 20 presents the ANOVA and Tukey HSD multiple-comparison analysis for sulphur penetration depth. The ANOVA results demonstrated that biomass type, pyrolysis temperature, replacement level, and their interaction effects significantly influenced sulphur penetration resistance (p < 0.05). The effect-size values further indicate that replacement level and pyrolysis temperature were the dominant factors governing sulphur ingress resistance.

**Table 20:** ANOVA and Tukey HSD multiple comparison analysis for sulphur penetration depth of montmorillonite-modified burnt red soil bricks incorporating PCG and PTG.

Factor / Comparison	Mean ± SD (mm)	COV (%)	95% Confidence Interval	df	F-Value	p-Value	η²	Significance
ANOVA RESULTS								
Biomass Type (A)	24.83 ± 6.44	25.94	22.12–27.54	1	29.47	<0.001	0.237	Significant
Pyrolysis Temperature (B)	24.65 ± 6.92	28.07	21.74–27.56	2	38.91	<0.001	0.308	Significant
Replacement Level (C)	25.12 ± 7.63	30.37	21.91–28.33	3	65.84	<0.001	0.435	Highly Significant
A × B	—	—	—	2	7.84	0.002	0.072	Significant
A × C	—	—	—	3	9.28	0.001	0.085	Significant
B × C	—	—	—	6	13.24	<0.001	0.121	Significant
A × B × C	—	—	—	6	5.76	0.006	0.056	Significant
TUKEY HSD MULTIPLE COMPARISONS								
PCG350−10 vs PCG350−15	—	—	2.10–6.30	—	—	0.002	—	Significant
PCG350−10 vs PCG300−10	—	—	6.20–11.80	—	—	<0.001	—	Highly Significant
PCG350−10 vs PCG500−10	—	—	5.10–10.90	—	—	<0.001	—	Highly Significant
PCG350−10 vs PTG300−10	—	—	2.80–7.20	—	—	<0.001	—	Highly Significant
5% vs 10% Replacement	—	—	2.40–6.40	—	—	0.005	—	Significant
10% vs 15% Replacement	—	—	2.00–5.20	—	—	0.009	—	Significant
10% vs 20% Replacement	—	—	11.80–18.20	—	—	<0.001	—	Highly Significant
DESCRIPTIVE STATISTICS AND GROUPING								
PCG350−10	11.0 ± 0.8	7.27	10.0–12.0	—	—	—	—	Group a
PCG350−15	15.0 ± 1.1	7.33	13.6–16.4	—	—	—	—	Group b
PTG300−10	16.0 ± 1.1	6.88	14.6–17.4	—	—	—	—	Group c
PTG300−15	18.0 ± 1.2	6.67	16.5–19.5	—	—	—	—	Group d
PCG350−5	19.0 ± 1.2	6.32	17.5–20.5	—	—	—	—	Group e
PCG500−10	19.0 ± 1.2	6.32	17.5–20.5	—	—	—	—	Group e
Control	35.0 ± 1.1	3.14	33.6–36.4	—	—	—	—	Group f

Among all investigated mixtures, PCG350−10 exhibited the lowest sulphur penetration depth (11.0 ± 0.8 mm) and was assigned to Group a, indicating the highest resistance to sulphur attack. Compared with the control specimen (35.0 ± 1.1 mm), this mixture reduced sulphur penetration by approximately 68.6%. Tukey HSD analysis confirmed that PCG350−10 was significantly superior to PCG350−15, PCG300−10, PCG500−10, and PTG300−10 (p < 0.05), demonstrating that the reduction in penetration depth was statistically significant.

The results further revealed that increasing the replacement level from 5% to 10% substantially improved sulphur resistance, whereas further increases to 15% and 20% resulted in progressively greater penetration depths. This trend suggests that moderate incorporation of pyrolyzed biomass improved matrix compactness and reduced the continuity of transport pathways. However, excessive replacement levels likely introduced additional pores and weak interparticle regions that facilitated sulphur ingress.

Pyrolysis temperature also exerted a significant influence on sulphur penetration. For both PCG and PTG mixtures, specimens produced at 350°C consistently exhibited lower penetration depths than those produced at 300°C and 500°C. This finding indicates that the intermediate pyrolysis temperature provided the most favorable balance between carbonization and microstructural refinement. Furthermore, PCG-modified bricks generally outperformed the corresponding PTG-modified bricks, demonstrating the greater effectiveness of pyrolyzed coffee grounds in enhancing resistance to sulphur attack [1–5].

Overall, the statistical analysis confirms that PCG350−10 was the optimum formulation for minimizing sulphur penetration and improving durability performance. The combined ANOVA and Tukey HSD results provide strong statistical evidence that the superior sulphur resistance of this mixture resulted from the synergistic interaction of biomass type, pyrolysis temperature, and replacement level.

## 4. Conclusions

This study investigated the effects of pyrolyzed coffee grounds (PCG) and pyrolyzed tobacco grounds (PTG), produced at 300°C, 350°C, and 500°C and incorporated at replacement levels of 5–20%, on the mechanical, durability, microstructural, and physicochemical performance of burnt red soil bricks containing 10% montmorillonite. Based on the experimental results, the following conclusions can be drawn:

1. Both PCG and PTG significantly influenced the engineering performance of burnt red soil bricks, with the magnitude of improvement depending on pyrolysis temperature and replacement level. Moderate incorporation levels generally enhanced performance, whereas excessive replacement (20%) resulted in reductions in both mechanical and durability properties.
2. Among all investigated mixtures, PCG350−10 consistently exhibited the best overall performance. The compressive, tensile, and flexural strengths increased from 8.5 MPa, 1.5 MPa, and 2.2 MPa for the control specimen to 11.8 MPa, 2.8 MPa, and 3.9 MPa, respectively, corresponding to improvements of approximately 38.8%, 86.7%, and 77.3%.
3. Durability performance was substantially improved through the incorporation of optimally pyrolyzed biomass. The PCG350−10 mixture reduced water, chloride, and sulphur penetration depths from 38 mm, 32 mm, and 35 mm in the control specimen to 12 mm, 10 mm, and 11 mm, respectively, representing reductions of approximately 68–69%.
4. FTIR analysis indicated progressive modification of oxygen-containing functional groups with increasing pyrolysis temperature. XRD results demonstrated the persistence of quartz as the dominant crystalline phase together with temperature-dependent variations in secondary mineral phases. DSC analysis revealed significant thermal transformations associated with dehydration and organic matter decomposition, while Raman spectroscopy confirmed increasing structural ordering of carbonaceous components following pyrolysis.
5. SEM observations revealed that the optimum mixtures developed a more compact and homogeneous microstructure with fewer visible pores and discontinuities than the control and high-replacement specimens. The improved microstructural integrity is consistent with the observed enhancement in both strength and resistance to fluid ingress.
6. The superior performance of the PCG350−10 mixture suggests that pyrolysis at 350°C produced a favorable balance between biomass stabilization and structural preservation. At this condition, the biomass-derived particles contributed to improved particle packing, reduced pore connectivity, and enhanced resistance to crack initiation and transport of aggressive agents.
7. Comparison between PCG and PTG mixtures demonstrated that coffee-ground-derived biochar generally provided greater improvements in both mechanical and durability properties than tobacco-ground-derived biochar under similar processing conditions, indicating differences in biomass composition and pyrolysis behavior.

Overall, the findings demonstrate that controlled pyrolysis of agricultural waste materials can provide an effective route for enhancing the performance of sustainable burnt red soil bricks while simultaneously promoting waste valorization and resource efficiency. Within the investigated range, the incorporation of 10% PCG pyrolyzed at 350°C is recommended as the optimum formulation for achieving a balanced combination of mechanical strength, durability, and microstructural stability.

The present study focused on laboratory-scale evaluation of brick performance. Future investigations should examine long-term field durability, freeze–thaw resistance, weathering performance, life-cycle assessment, and large-scale manufacturing feasibility to further validate the practical application of pyrolyzed biomass-modified burnt red soil bricks.

## Supporting information

S1 Fig Graphical abstract. (TIFF)

S2 Fig Research methodology. (TIFF)

## Abbreviations

PCG: Pyrolyzed Coffee Grounds
PTG: Pyrolyzed Tobacco Grounds
SCG: Spent Coffee Grounds
STG: Spent Tobacco Grounds
MMT: Montmorillonite
FTIR: Fourier Transform Infrared; Spectroscopy
XRD: X-ray Diffraction
SEM: Scanning Electron Microscopy
DSC: Differential Scanning Calorimetry
ANOVA: Analysis of Variance
